# Supplementary material for: Conserved Patterns in Developmental Processes and Phases, Rather than Genes, Unite the Highly Divergent Bilateria
Source: Life (Basel). 2020 Sep 6;10(9):182. doi: 10.3390/life10090182 (PMC7555945; doi:10.3390/life10090182)
Supplement: Supplementary file 1 [file life-10-00182-s001.pdf]

Supplementary Figures and Tables

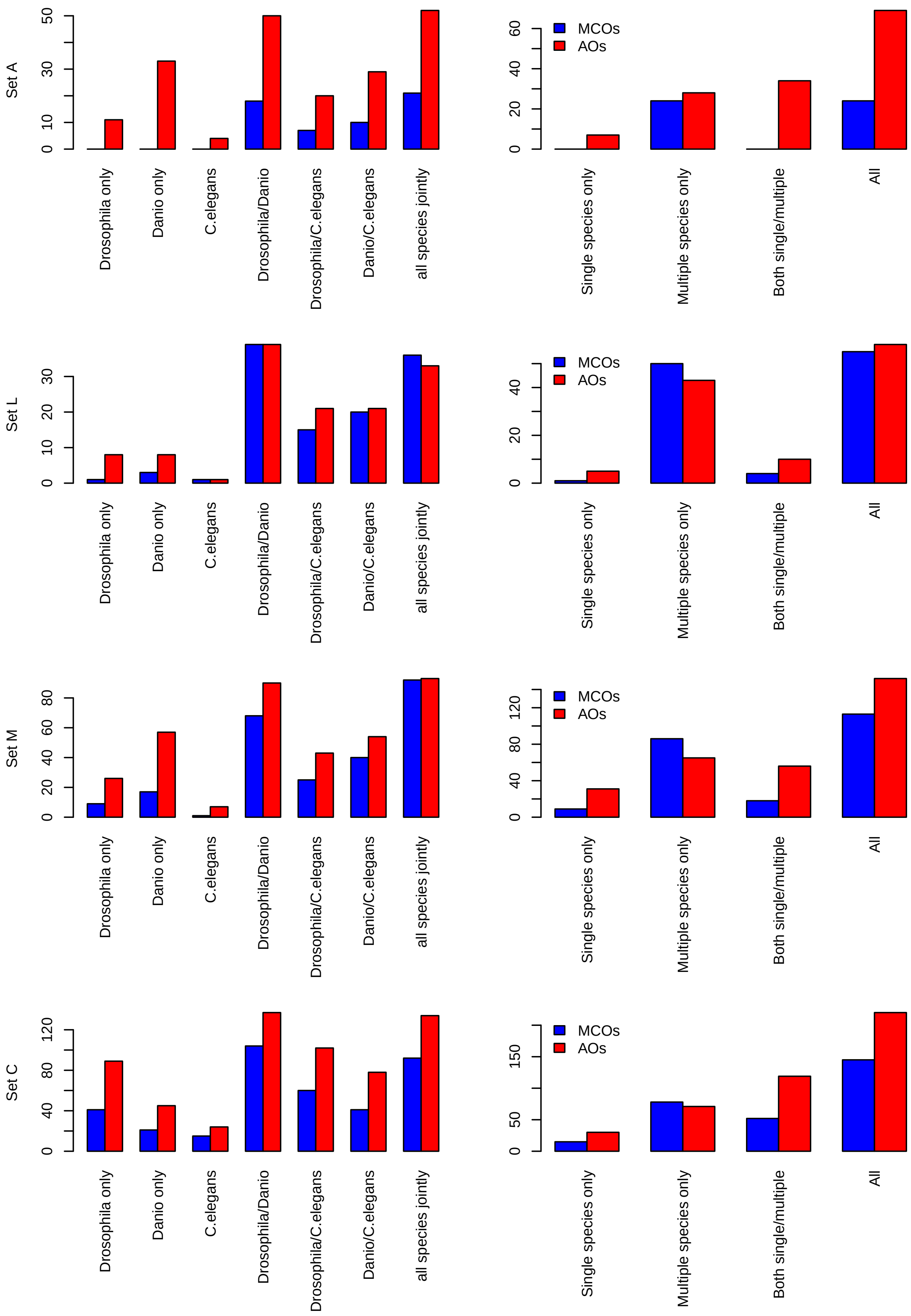

Figure S1. Supplementary Figure related to Figure 4. MSGEA vs. single species enrichment analysis for clusters of orthologous proteins (COPs). Number of significant terms shown for each of the four sets A, L, M, and C (cf. Figure 1).

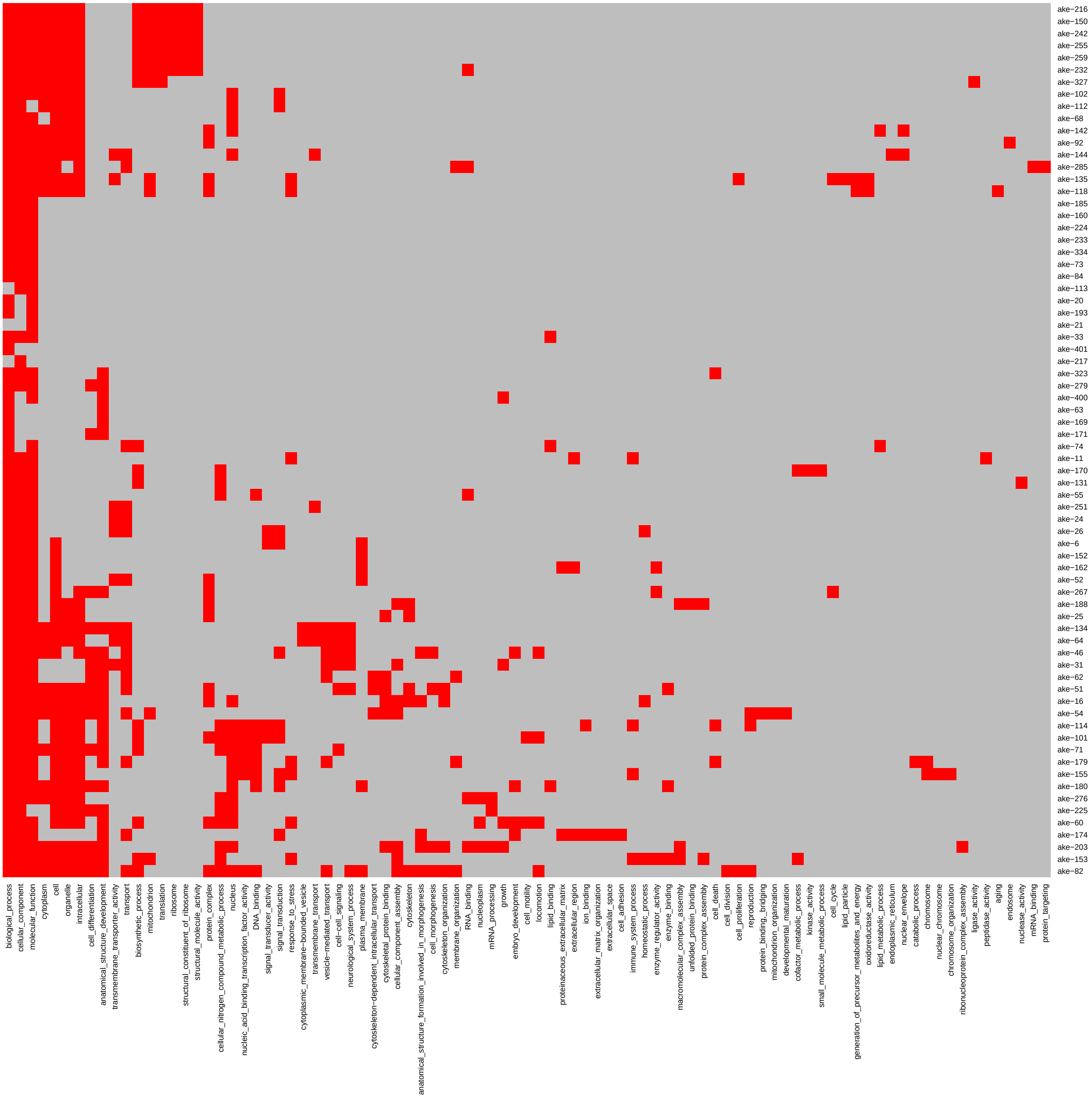

Figure S2. Supplementary Figure related to Figure 5. Absence/presence map of GO-slim terms. x-axis: GO-slim terms (including all GO levels); right y-axis: internal COP IDs.

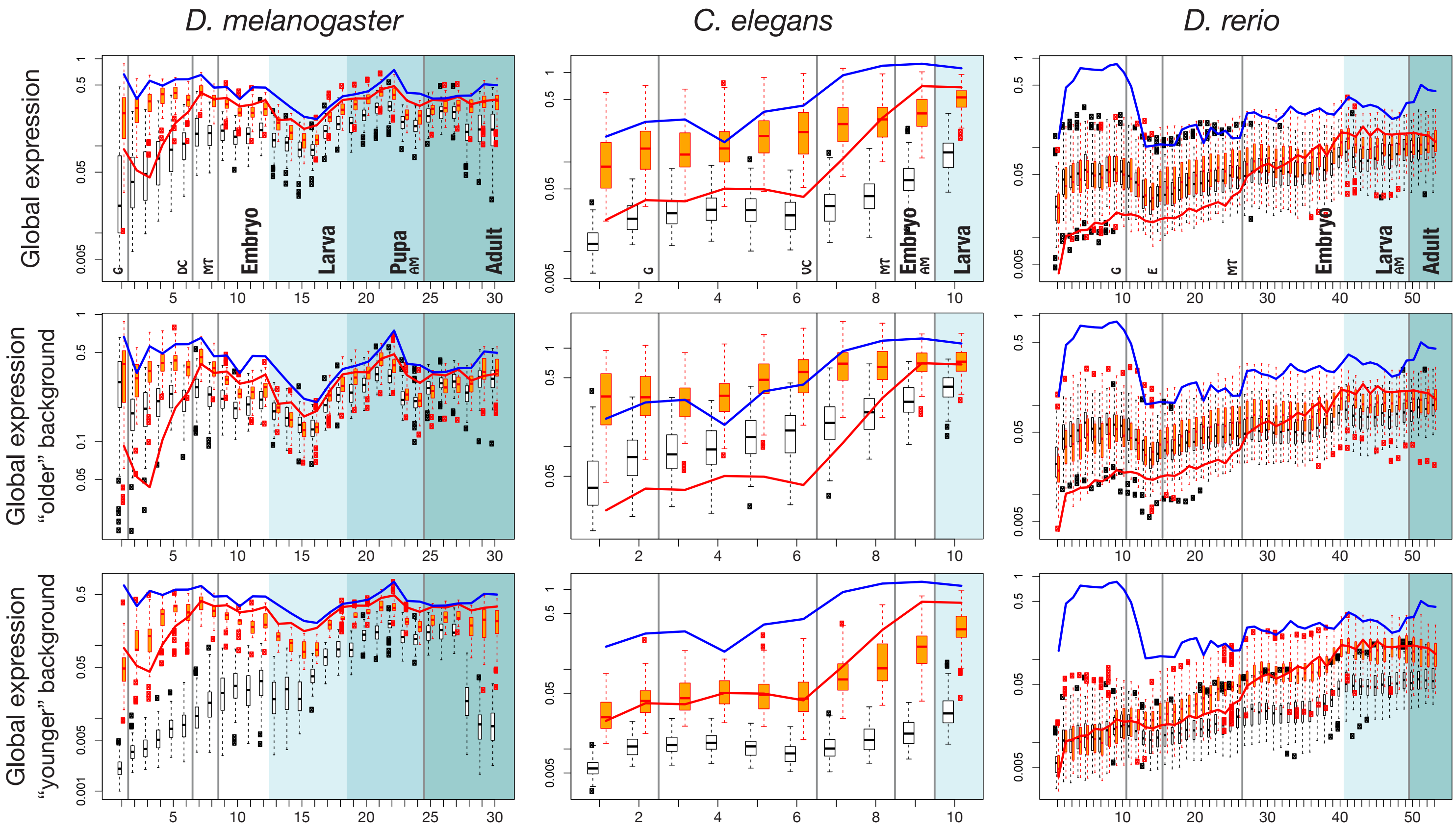

Figure S3. Supplementary Figure related to Figure 3. Expression profiles with background distribution based on medians, instead of means. Color code and other labels as in Figure 5.

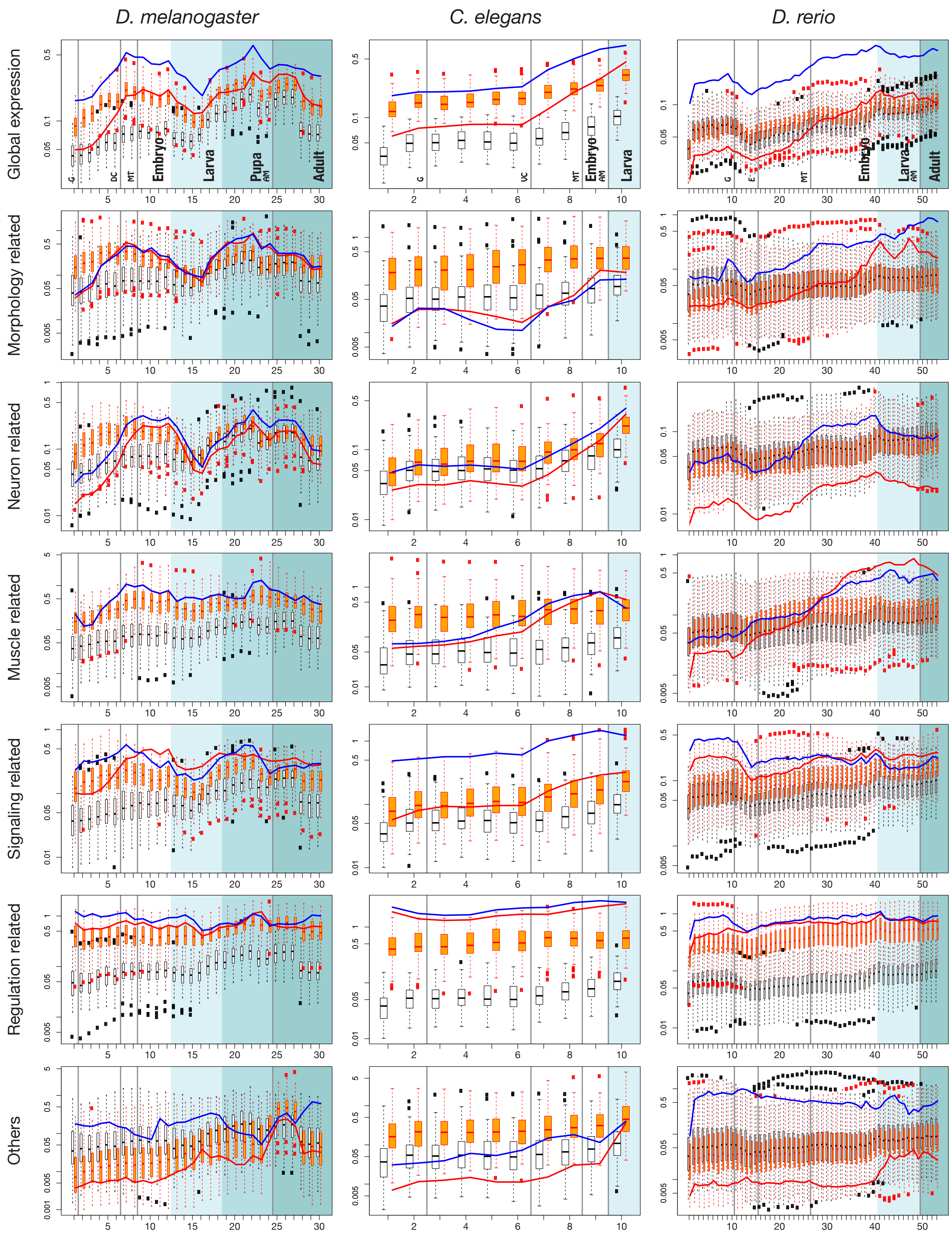

Figure S4. Supplementary Figure related to Figure 3. Expression profiles of set L' genes by functional classes. Background distributions based on mean expression values. Color code and other labels as in Figure 5. *D.m.*, 'muscle': there are no paralogs; therefore, only the blue line (for MCOs) is displayed.

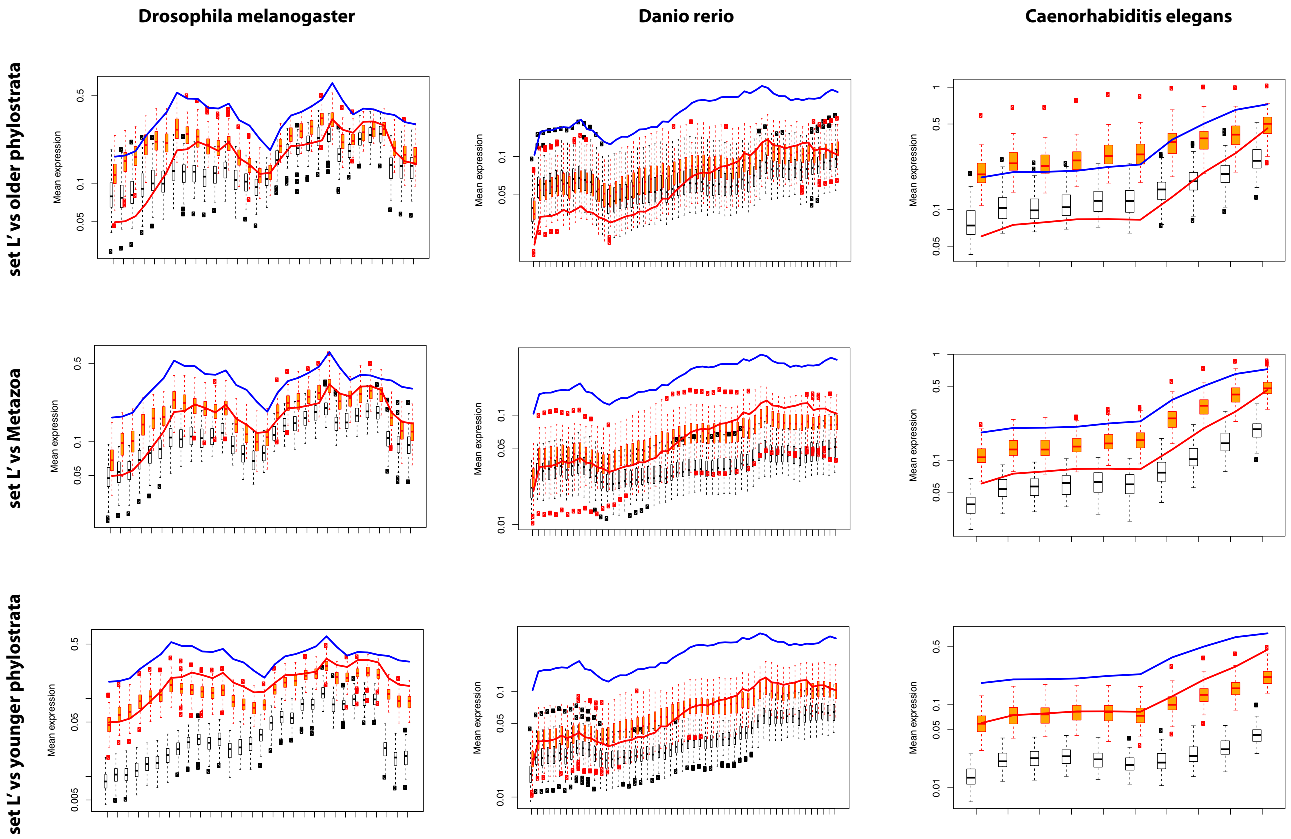

Figure S5. Supplementary Figure related to Figure 3. Expression profiles of set L' genes compared to different background distributions, including all phylostratigraphically older and younger genes at the top and bottom, and all Metazoan genes in the middle. Background distributions based on mean expression values. Color code and other labels as in Figure 5.
.

Table S1. Supplementary Table related to Figure 1. Distribution of losses in set L.

**number of clusters**	**species affected**	**lineage affected**
[33]	[APL]	[APL only]
31	APL*, CEL	CEL only
22	APL*, STR	STR only
8	APL*, HEL	HEL only
6	APL*, ANO, DRO	insects
6	APL*, ANO	ANO only
4	APL*, MUS	MUS only
4	APL*, LOT	LOT only
4	APL*, CAP	CAP only
3	APL*, DRO	DRO only
2	APL*, HEL, CAP	annelids
1	ANO	ANO only
1	-	-

*due to the incomplete assembly and annotation status of the *A. californica* genome, APL was ignored when determining the number of losses. When *A. californica* is included only one cluster contains orthologs from all species (last line). Omitting *A. californica*, we find 33 clusters which contain orthologs from nine species (first line). In the remaining 91 clusters in set L an ortholog from at least one species is missing. Most losses (31 of 91) are *C. elegans*-specific (second line).

Table S2. Supplementary Table with annotation of x-axis tick marks in Figure 5, Figure S3 and Figure S4 according to [75] (*D. melanogaster*), [124] (*C. elegans*) and [48] (*D. rerio*).

D. rerio

bckgr-color	tickmark	devel. stage
white	1	egg 0min
	2	zygote 15min
	3	cleavage 45min
	4	cleavage 1h15min
	5	cleavage 1h45min
	6	blastula 2h15min
	7	blastula 2h45min
	8	blastula 3h20min
	9	blastula 4h
	10	blastula 4h40min
	11	gastrula 5h20min
	12	gastrula 6h
	13	gastrula 7h
	14	gastrula 8h
	15	gastrula 9h
	16	gastrula 10h
	17	segmentation 10h20min
	18	segmentation 11h
	19	segmentation 11h40min
	20	segmentation 12h
	21	segmentation 13h
	22	segmentation 14h
	23	segmentation 15h
	24	segmentation 16h
	25	segmentation 17h
	26	segmentation 18h
	27	segmentation 19h
	28	segmentation 20h
	29	segmentation 21h
	30	segmentation 22h
	31	segmentation 23h
	32	pharyngula 1d1h
	33	pharyngula 1d3h
	34	pharyngula 1d6h
	35	pharyngula 1d10h
	36	pharyngula 1d14h
	37	pharyngula 1d18h
	38	hatching 2d
	39	hatching 2d12h
	40	hatching 3d
light grey	41	larva 4d
	42	larva 6d
	43	larva 8d
	44	larva 10d
	45	larva 14d
	46	larva 18d
	47	larva 24d
	48	larva 30d
	49	larva 40d
dark grey	50	juvenile 45d
	51	juvenile 55d
	52	juvenile 65d
	53	juvenile 80d

D. melanogaster

bckgr-color	tickmark	devel. stage
white	1	embryos 0-2hr
	2	embryos 2-4hr
	3	embryos 4-6hr
	4	embryos 6-8hr
	5	embryos 8-10hr
	6	embryos 10-12hr
	7	embryos 12-14hr
	8	embryos 14-16hr
	9	embryos 16-18hr
	10	embryos 18-20hr
	11	embryos 20-22hr
	12	embryos 22-24hr
light grey	13	L1 larvae
	14	L2 larvae
	15	L3 larvae 12hr post molt
	16	L3 larvaePS 1-2
	17	L3 larvaePS 3-6
	18	L3 larvaePS 7-9
grey	19	white prepupae
	20	WPP 12hr
	21	WPP 24hr
	22	pupae WPP 2d
	23	pupae WPP 3d
	24	pupae WPP 4d
dark grey	25	adult male 1d
	26	adult male 30d
	27	adult male 5d
	28	adult female 1d
	29	adult female 5d
	30	adult female 30d

C. elegans

bckgr-color	tickmark	devel. stage
white	1	stage1
	2	stage2
	3	stage3
	4	stage4
	5	stage5
	6	stage6
	7	stage7
	8	stage8
	9	stage9
light grey	10	stage10

Table S3. Supplementary Table related to Figure S3 and Figure S4. Comparison of developmental stages among *Drosophila*, *Caenorhabditis*, and *Danio*

D. rerio	&		&		&				&	C.elegans	&		&		&				&	Drosophila				
Tick marks	&	Point of time	&	Stage	&	Description			&	Tick marks	&	Point of time at 20ºC	&	Stage	&	Description			&	Tick marks	Point of time at 25ºC	Description		
1	&	0 min	&	1-cell	&	Zygote period	:	Cytoplasma streams toward animal pole to form the blastodis	&	.	&		&		&				&	1	0-2 h	Cleavage	:	Mitotic cycles; cell divison
2	&	15 min	&		&	Zygote period			&	1	&	20-40 min	&	4-cells	&	Zygote to embryonic founder cell			&					
3	&	45 min	&	2-cells	&	Cleavage period	:	2x2 array of blastomeres	&	2	&		&	14-cells	&	Zygote to embryonic founder cell	:	E-division	&					
4	&	1h 15 min	&	8-cells	&	Cleavage period	:	2x4 array of blastomeres	&	.	&		&		&				&					
5	&	1h 45min	&	32-cells	&	Cleavage period	:	2 regular tiers of blastomeres	&	.	&		&		&				&					
6	&	2h 15 min	&	128-cells	&	Blastula period	:	5 blastomere tiers	&	.	&		&		&				&					
7	&	2h 45min	&	512-cells	&	Blastula period	:	9 blastomeres tiers	&	.	&		&		&				&					
8	&	3h 20 min	&	1k-cells	&	Blastula period	:	>11 tiers of blastomeres; beginning of blastodisc flattening	&	.	&		&		&				&					
9	&	4h	&	Sphere	&	Blastula period	:	Spherical shape	&	.	&		&		&				&					
10	&	4h 40 min	&	30%-epiboly	&	Blastula period	:	Blastoderm an inverted cup of uniform thickness	&	3	&		&	24-cells	&	Zygote to embryonic founder cell	:	4th division of the AB lineage	&		2:10-2:50 h	Blastoderm		
	&		&		&				&		&		&		&				&					
11	&	5h 20min	&	50%-epiboly	&	Gastrula period	:	Blastoderm remains uniform in thickness	&	.	&	100-290 min	&	26-cells	&	Cell division and gastrulation	:	Beginning of gastrulation	&	2	2-4 h	Gastrulation	:	Cellularization of the blastoderm; form mesoderm and endoderm
12	&	6h	&	Shield	&	Gastrula period	:	Embryonic shield visible from animal pol	&	4	&		&	44-cells	&	Cell division and gastrulation	:	5th division of the AB lineage	&	3	4-6 h	Germ band	:	Segmentation of neuroblasts; head formation starts, postblastoderm mitosis
13	&	7h	&		&	Gastrula period			&	5	&		&	68-cells	&	Cell division and gastrulation	:	6th division of the AB lineage	&	4	6-8 h	Germ band	:	End of neuroblast formation; epidermal parasegmentation evident
14	&	8h	&	75%-epiboly	&	Gastrula period	:	Dorsal side distinctly thicker	&	6	&	145-150 min	&	86-cell	&	Cell division and gastrulation	:	7th division of the AB lineage	&	5	8-10 h			
15	&	9h	&	90%-epiboly	&	Gastrula period	:	brain rudiment thickened; notochord rudiment distinct from se	&	.	&		&		&				&					
16	&	10h	&	100%-epiboly	&	Gastrula period	:	Enveloping layer closes; notochord distinct from neural keel; t	&	7	&	290-350 min	&	Ventral closure	&	Cell division and gastrulation	:	Epidermal morphogenesis; dorsal epiderma	&	6	10-12 h	Dorsal closure	:	Head involution begins; end of germ band retraction; CNS and PNS differentation
17	&	10h 20min	&	1-somite	&	Segmentation period			&	.	&		&		&				&	7	12-14 h	Differentiation	:	End of dorsal closure; head involution; discs invagination
18	&	11h	&		&	Segmentation period			&	.	&		&		&				&	8	14-16 h	Differentiation	:	Shortening of the ventral nerv cord; advanced denticles visible
19	&	11h 40min	&	5-somite	&	Segmentation period	:	Polster prominent; optical vesicle	&	.	&		&		&				&					
20	&	12h	&		&	Segmentation period			&	.	&		&		&				&					
21	&	13h	&		&	Segmentation period			&	8	&	390-420 min	&	560-cells; Comma Stage	&	Differentiation of cells			&					
22	&	14h	&		&	Segmentation period			&	.	&		&		&				&					
23	&	15h	&		&	Segmentation period			&	.	&		&		&				&					
24	&	16h	&	14-somite	&	Segmentation period	:	EL=0.9mm; brain neuromeres	&	.	&		&		&				&					
25	&	17h	&		&	Segmentation period			&	.	&		&		&				&					
26	&	18h	&		&	Segmentation period			&	.	&		&		&				&					
27	&	19h	&	20-somite	&	Segmentation period	:	EL=1.4mm; hindbrain neuromeres prominent; muscular twitc	&	9	&	440-480 min	&	560-cells; Movement	&	Differentiation of cells	:	First muscle twitches are visible	&	9	16-18 h	Differentiation	:	The tracheal t (paper cite; The Development of Motor Coordination in Drosophila Embryos, 2008, Sarah Crisp)
28	&	20h	&		&	Segmentation period			&	.	&		&		&				&	10	18-20 h	Differentiation	:	The tracheal tree fills with air; retraction of the ventral cord continues
29	&	21h	&		&	Segmentation period			&	.	&		&		&				&	11	20-22 h	Hatch from egg	:	First instar larva
30	&	22h	&	26-somite	&	Segmentation period	:	EL=1.6mm; midbrain-hindbrain boundary; blood islands	&	.	&		&		&				&	12	22-24 h	Hatch from egg	:	First instar larva
31	&	23h	&		&	Segmentation period			&	.	&		&		&				&	13	L1 larvae	Methamorphosis	:	First larval stage ; end with first larval molt; mouth hooks have one tooth
32	&	1d 1h	&	Prim-5	&	Pharyngula period	:	EL=1.9mm; red blood cells on yolk; heartbeat	&	.	&		&		&				&	14	L2 larvae	Methamorphosis	:	Second larval stage; ends at the second larval molt; larvae are actively feeding and crawling; mouth hooks have two or three teeth
33	&	1d3h	&		&	Pharyngula period			&	.	&		&		&				&	15	L3 larvae 12h post-molt	Methamorphosis	:	Early third larval stage
34	&	1d 6h	&	Prim-15	&	Pharyngula period	:	EL=2.5mm; early touch reflex; retina pigmented	&	.	&		&		&				&	16	L3 larvae dark blue gut PS (1-2)	Methamorphosis	:	Third larval stage; larva wanders
35	&	1d 10h	&		&	Pharyngula period			&	.	&		&		&				&	17	L3 larvae light blue gut PS (3-6)	Methamorphosis	:	Third larval stage; larva wanders out of food
36	&	1d 14h	&		&	Pharyngula period			&	.	&		&		&				&	18	L3 larvae clear gut PS (7-9)	Methamorphosis	:	Third instar larva stops crawling
37	&	1d 18h	&	High-pec	&	Pharyngula period	:	EL=2.9mm; complete dorsal stripe; rudiments of pectoral fins	&	.	&	620-800 min	&	560-cells	&	Late embyro	:	Fully-elongated embryo	&	19	WPP+12h	Methamorphosis	:	White prepupae
	&		&		&				&		&		&		&				&	20	WPP+18h	Methamorphosis	:	White prepupae
	&		&		&				&		&		&		&				&	21	WPP+24, pupae	Methamorphosis	:	White prepupae
	&		&		&				&		&		&		&				&	22	WPP+2days, pupae	Methamorphosis	:	White prepupae
	&		&		&				&		&		&		&				&	23	WPP+3 days, pupae	Methamorphosis	:	White prepupae
	&		&		&				&		&		&		&				&					
38	&	2d	&	Long-pec	&	Hatching period	:	EL=3.1mm; elongated pectoral fin buds	&	.	&		&		&	Post-embryonic	:	after hatching	&	24	WPP+4 days, adult male			
39	&	2d 12h	&		&	Hatching period			&	.	&		&		&				&	25	Eclosion+1 day, adult male			
40	&	3d	&	Protruding-mouth	&	Hatching period	:	3.5mm total body length	&	.	&		&		&				&	26	Eclosion+5 days, adult male			
41	&	4d	&		&	Larva			&	10	&	14-25.5 h at 25 °C	&	First stage larva	&	Further development of nervous and reproduction system			&	27	Eclosion+30 days, adult male			
42	&	6d	&		&	Larva			&	.	&		&		&				&	28	Eclosion+1 day, adult female			
43	&	8d	&		&	Larva			&	.	&		&		&				&	29	Eclosion+5 days, adult female			
44	&	10d	&		&	Larva			&	.	&		&		&				&	30	Eclosion+30 days, adult female			
45	&	14d	&		&	Larva			&	.	&		&		&				&					
46	&	18d	&		&	Larva			&	.	&		&		&				&					
47	&	24d	&		&	Larva			&	.	&		&		&				&					
48	&	30d	&		&	Larva			&	.	&		&		&				&					
49	&	40d	&		&	Larva			&	.	&		&		&				&					
50	&	45d	&		&	Juvenile			&	.	&		&		&				&					
51	&	55d	&		&	Juvenile			&	.	&		&		&				&					
52	&	65d	&		&	Juvenile			&	.	&		&		&				&					
[illegible]	&	[illegible]	&		&	[illegible]			&		&		&		&				&					

Table S4. Supplementary Table related to Figures 4 and 5. GO terms (of level ≥ 4) identified in 85 COPs of set L'. COPs ake-68, ake-73, ake-185, ake-217 and ake-224 did not contain GO terms of level ≥ 4 and do therefore not occur in this table.

GO-ID	classification	GO description	internal COP-ID (ake-###)
GO:0000122	regulation	negative regulation of transcription from RNA polymerase II promoter	114, 37, 82
GO:0000188	signalling	inactivation of MAPK activity	3
GO:0000226	muscle	microtubule cytoskeleton organization	120, 51
GO:0000278	morphology	mitotic cell cycle	135, 267
GO:0000280	signalling	nuclear division	16
GO:0000302	signalling	response to reactive oxygen species	118
GO:0000381	regulation	regulation of alternative nuclear mRNA splicing, via spliceosome	276
GO:0000387	signalling	spliceosomal snRNP assembly	203
GO:0000422	regulation	mitochondrion degradation	193
GO:0001525	morphology	angiogenesis	6
GO:0001653	signalling	peptide receptor activity	6
GO:0001666	signalling	response to hypoxia	135, 193
GO:0001708	morphology	cell fate specification	82
GO:0001709	morphology	cell fate determination	32
GO:0001751	neuron	compound eye photoreceptor cell differentiation	180, 32
GO:0001754	neuron	eye photoreceptor cell differentiation	64, 82
GO:0001756	morphology	somitogenesis	2, 37, 6
GO:0001946	morphology	lymphangiogenesis	82
GO:0001947	muscle	heart looping	3
GO:0001964	signalling	startle response	37, 55
GO:0002009	morphology	morphogenesis of an epithelium	120, 16, 171, 92
GO:0002074	muscle	extraocular skeletal muscle development	71
GO:0002119	morphology	nematode larval development	114, 131, 150, 155, 16, 171, 174, 180, 18, 203, 216, 232, 242, 255, 259, 279, 46, 71, 82, 92, 97
GO:0002121	signalling	inter-male aggressive behavior	52, 55
GO:0002385	signalling	mucosal immune response	155
GO:0003140	morphology	determination of left/right asymmetry in lateral mesoderm	3
GO:0003146	muscle	heart jogging	3
GO:0003707	signalling	steroid hormone receptor activity	114
GO:0003810	signalling	protein-glutamine gamma-glutamyltransferase activity	3
GO:0003917	regulation	DNA topoisomerase type I activity	3
GO:0003918	regulation	DNA topoisomerase (ATP-hydrolyzing) activity	3
GO:0003924	signalling	GTPase activity	3
GO:0003951	regulation	NAD+ kinase activity	170
GO:0003954	regulation	NADH dehydrogenase activity	118
GO:0004091	signalling	carboxylesterase activity	51
GO:0004129	signalling	cytochrome-c oxidase activity	135
GO:0004190	signalling	aspartic-type endopeptidase activity	113
GO:0004221	signalling	ubiquitin thiolesterase activity	3
GO:0004252	signalling	serine-type endopeptidase activity	11
GO:0004308	signalling	exo-alpha-sialidase activity	3
GO:0004386	signalling	helicase activity	162
GO:0004518	signalling	nuclease activity	131
GO:0004519	signalling	endonuclease activity	131
GO:0004521	signalling	endoribonuclease activity	131
GO:0004553	signalling	hydrolase activity, hydrolyzing O-glycosyl compounds	51
GO:0004556	signalling	alpha-amylase activity	27
GO:0004672	signalling	protein kinase activity	144
GO:0004674	signalling	protein serine/threonine kinase activity	144
GO:0004725	signalling	protein tyrosine phosphatase activity	3
GO:0004812	regulation	aminoacyl-tRNA ligase activity	160, 32
GO:0004842	signalling	ubiquitin-protein ligase activity	2

GO-ID	classification	GO description	internal COP-ID (ake-###)
GO:0004887	signalling	thyroid hormone receptor activity	114
GO:0004888	signalling	transmembrane signaling receptor activity	6
GO:0004930	signalling	G-protein coupled receptor activity	3, 6
GO:0004948	signalling	calcitonin receptor activity	6
GO:0004991	signalling	parathyroid hormone receptor activity	6
GO:0005085	signalling	guanyl-nucleotide exchange factor activity	63
GO:0005086	signalling	ARF guanyl-nucleotide exchange factor activity	3
GO:0005089	signalling	Rho guanyl-nucleotide exchange factor activity	63
GO:0005184	neuron	neuropeptide hormone activity	6
GO:0005216	signalling	ion channel activity	144, 3, 52
GO:0005253	signalling	anion channel activity	19
GO:0005254	signalling	chloride channel activity	19, 52
GO:0005261	signalling	cation channel activity	144
GO:0005262	signalling	calcium channel activity	134
GO:0005267	signalling	potassium channel activity	144
GO:0005277	signalling	acetylcholine transmembrane transporter activity	251
GO:0005278	signalling	acetylcholine:hydrogen antiporter activity	251
GO:0005326	neuron	neurotransmitter transporter activity	31
GO:0005430	neuron	synaptic vesicle amine transmembrane transporter activity	64
GO:0005786	signalling	signal recognition particle, endoplasmic reticulum targeting	285
GO:0005802	signalling	trans-Golgi network	18
GO:0005913	signalling	cell-cell adherens junction	180
GO:0005975	signalling	carbohydrate metabolic process	27, 51
GO:0006030	morphology	chitin metabolic process	2
GO:0006120	regulation	mitochondrial electron transport, NADH to ubiquinone	118
GO:0006123	regulation	mitochondrial electron transport, cytochrome c to oxygen	135
GO:0006171	signalling	cAMP biosynthetic process	6
GO:0006184	signalling	GTP catabolic process	3
GO:0006200	regulation	ATP catabolic process	162, 3
GO:0006260	regulation	DNA replication	3
GO:0006265	regulation	DNA topological change	3
GO:0006281	regulation	DNA repair	33
GO:0006306	regulation	DNA methylation	6
GO:0006351	regulation	transcription, DNA-dependent	101, 2, 3, 37, 71
GO:0006355	regulation	regulation of transcription, DNA-dependent	101, 114, 2, 32, 37, 3, 71, 82
GO:0006357	regulation	regulation of transcription from RNA polymerase II promoter	101, 37
GO:0006364	regulation	rRNA processing	401
GO:0006396	regulation	RNA processing	225
GO:0006397	signalling	mRNA processing	203, 276
GO:0006412	regulation	translation	150, 216, 232, 242, 255, 259, 327, 97
GO:0006418	regulation	tRNA aminoacylation for protein translation	160, 32
GO:0006450	regulation	regulation of translational fidelity	327
GO:0006457	signalling	protein folding	112
GO:0006468	signalling	protein phosphorylation	144
GO:0006470	signalling	protein dephosphorylation	3
GO:0006486	signalling	protein glycosylation	2, 3
GO:0006508	signalling	proteolysis	113, 3
GO:0006511	signalling	ubiquitin-dependent protein catabolic process	3
GO:0006614	regulation	SRP-dependent cotranslational protein targeting to membrane	285
GO:0006629	signalling	lipid metabolic process	142
GO:0006694	signalling	steroid biosynthetic process	74
GO:0006754	regulation	ATP biosynthetic process	3
GO:0006811	signalling	ion transport	144, 3, 52
GO:0006812	signalling	cation transport	3

GO-ID	classification	GO description	internal COP-ID (ake-###)
GO:0006813	signalling	potassium ion transport	144, 26
GO:0006814	signalling	sodium ion transport	24, 3
GO:0006820	signalling	anion transport	19
GO:0006821	signalling	chloride transport	19, 52
GO:0006826	signalling	iron ion transport	3
GO:0006836	neuron	neurotransmitter transport	101, 251, 31, 64
GO:0006855	signalling	drug transmembrane transport	251, 64
GO:0006869	signalling	lipid transport	74
GO:0006874	signalling	cellular calcium ion homeostasis	5
GO:0006879	signalling	cellular iron ion homeostasis	3
GO:0006884	signalling	cell volume homeostasis	19
GO:0006885	signalling	regulation of pH	3
GO:0006886	signalling	intracellular protein transport	14
GO:0006898	signalling	receptor-mediated endocytosis	102, 120, 131, 150, 232, 242, 259, 279, 46, 92
GO:0006911	signalling	phagocytosis, engulfment	52, 62, 82
GO:0006914	signalling	autophagy	193
GO:0006915	signalling	apoptotic process	3
GO:0006952	signalling	defense response	152, 2
GO:0007005	regulation	mitochondrion organization	5
GO:0007015	neuron	actin filament organization	120
GO:0007021	muscle	tubulin complex assembly	188
GO:0007026	muscle	negative regulation of microtubule depolymerization	25
GO:0007030	signalling	Golgi organization	135
GO:0007165	signalling	signal transduction	101, 112, 174, 180, 2, 3, 6
GO:0007166	signalling	cell surface receptor signaling pathway	6
GO:0007173	signalling	epidermal growth factor receptor signaling pathway	37
GO:0007186	signalling	G-protein coupled receptor signaling pathway	3, 6
GO:0007218	neuron	neuropeptide signaling pathway	6
GO:0007219	signalling	Notch signaling pathway	37, 46
GO:0007265	signalling	Ras protein signal transduction	180
GO:0007276	signalling	gamete generation	171
GO:0007281	morphology	germ cell development	276
GO:0007287	signalling	Nebenkern assembly	54
GO:0007297	signalling	ovarian follicle cell migration	32
GO:0007304	morphology	chorion-containing eggshell formation	32
GO:0007349	signalling	cellularization	46
GO:0007368	morphology	determination of left/right symmetry	3
GO:0007391	morphology	dorsal closure	46
GO:0007398	morphology	ectoderm development	37
GO:0007399	neuron	nervous system development	16, 37, 46, 51, 82
GO:0007400	neuron	neuroblast fate determination	82
GO:0007402	neuron	ganglion mother cell fate determination	82
GO:0007405	neuron	neuroblast proliferation	82
GO:0007406	neuron	negative regulation of neuroblast proliferation	82
GO:0007409	neuron	axonogenesis	16, 203, 51, 82
GO:0007411	neuron	axon guidance	82
GO:0007416	neuron	synapse assembly	82
GO:0007417	neuron	central nervous system development	82
GO:0007419	neuron	ventral cord development	174, 82
GO:0007420	neuron	brain development	3, 82
GO:0007422	neuron	peripheral nervous system development	32, 37, 82
GO:0007423	morphology	sensory organ development	82
GO:0007424	morphology	open tracheal system development	101
GO:0007426	morphology	tracheal outgrowth, open tracheal system	180
GO:0007427	signalling	epithelial cell migration, open tracheal system	101
GO:0007443	morphology	Malpighian tubule morphogenesis	174
GO:0007465	neuron	R7 cell fate commitment	82
GO:0007476	morphology	imaginal disc-derived wing morphogenesis	180
GO:0007498	morphology	mesoderm development	174, 37, 5

GO-ID	classification	GO description	internal COP-ID (ake-###)
GO:0007507	muscle	heart development	16, 2, 60
GO:0007517	muscle	muscle organ development	16, 71
GO:0007519	muscle	skeletal muscle tissue development	16, 71
GO:0007527	muscle	adult somatic muscle development	169
GO:0007528	neuron	neuromuscular junction development	203, 51
GO:0007552	morphology	metamorphosis	114
GO:0007553	morphology	regulation of ecdysteroid metabolic process	114
GO:0007591	morphology	molting cycle, chitin-based cuticle	114
GO:0007606	signalling	sensory perception of chemical stimulus	3
GO:0007616	neuron	long-term memory	22
GO:0007619	signalling	courtship behavior	82
GO:0008026	regulation	ATP-dependent helicase activity	162
GO:0008036	signalling	diuretic hormone receptor activity	6
GO:0008045	neuron	motor axon guidance	46
GO:0008049	signalling	male courtship behavior	6
GO:0008081	signalling	phosphoric diester hydrolase activity	33
GO:0008083	signalling	growth factor activity	3
GO:0008088	neuron	axon cargo transport	51, 62
GO:0008101	signalling	decapentaplegic signaling pathway	32
GO:0008104	signalling	protein localization	82
GO:0008137	regulation	NADH dehydrogenase (ubiquinone) activity	118
GO:0008138	signalling	protein tyrosine/serine/threonine phosphatase activity	3
GO:0008168	signalling	methyltransferase activity	6
GO:0008188	neuron	neuropeptide receptor activity	6
GO:0008233	signalling	peptidase activity	3
GO:0008234	signalling	cysteine-type peptidase activity	3
GO:0008284	signalling	positive regulation of cell proliferation	32
GO:0008285	signalling	negative regulation of cell proliferation	82
GO:0008293	signalling	torso signaling pathway	180
GO:0008355	neuron	olfactory learning	51
GO:0008356	morphology	asymmetric cell division	82
GO:0008373	signalling	sialyltransferase activity	3
GO:0008380	regulation	RNA splicing	203, 276
GO:0008417	signalling	fucosyltransferase activity	2
GO:0008504	signalling	monoamine transmembrane transporter activity	64
GO:0008508	signalling	bile acid:sodium symporter activity	24
GO:0008582	neuron	regulation of synaptic growth at neuromuscular junction	51
GO:0008587	morphology	imaginal disc-derived wing margin morphogenesis	37
GO:0008595	morphology	anterior/posterior axis specification, embryo	180
GO:0008898	signalling	homocysteine S-methyltransferase activity	2
GO:0009303	regulation	rRNA transcription	131
GO:0009306	signalling	protein secretion	46
GO:0009651	signalling	response to salt stress	155
GO:0009792	morphology	embryo development ending in birth or egg hatching	118, 131, 150, 155, 16, 169, 174, 193, 203, 21, 216, 232, 242, 255, 259, 276, 323, 334, 46, 51, 71, 92, 97
GO:0009881	signalling	photoreceptor activity	26
GO:0009966	signalling	regulation of signal transduction	180
GO:0009996	morphology	negative regulation of cell fate specification	32
GO:0010001	neuron	glial cell differentiation	82
GO:0010171	morphology	body morphogenesis	114, 120, 16, 18, 71
GO:0010466	signalling	negative regulation of peptidase activity	162
GO:0010468	signalling	regulation of gene expression	401
GO:0010629	signalling	negative regulation of gene expression	112, 37
GO:0010866	signalling	regulation of triglyceride biosynthetic process	155
GO:0010867	signalling	positive regulation of triglyceride biosynthetic process	142
GO:0014036	neuron	neural crest cell fate specification	3

GO-ID	classification	GO description	internal COP-ID (ake-###)
GO:0014812	muscle	muscle cell migration	60
GO:0015077	signalling	monovalent inorganic cation transmembrane transporter activity	3
GO:0015143	signalling	urate transmembrane transporter activity	21
GO:0015238	signalling	drug transmembrane transporter activity	251, 64
GO:0015269	signalling	calcium-activated potassium channel activity	26
GO:0015297	signalling	antiporter activity	3
GO:0015299	signalling	solute:hydrogen antiporter activity	3
GO:0015385	signalling	sodium:hydrogen antiporter activity	3
GO:0015662	regulation	ATPase activity, coupled to transmembrane movement of ions, phosphorylative mechanism	3
GO:0015672	signalling	monovalent inorganic cation transport	144, 3
GO:0015721	signalling	bile acid and bile salt transport	24
GO:0015842	neuron	synaptic vesicle amine transport	64
GO:0015844	signalling	monoamine transport	64
GO:0015872	signalling	dopamine transport	64
GO:0015893	signalling	drug transport	251, 64
GO:0015934	signalling	large ribosomal subunit	232
GO:0015992	signalling	proton transport	135, 3
GO:0016057	neuron	regulation of membrane potential in photoreceptor cell	26
GO:0016070	regulation	RNA metabolic process	55
GO:0016079	neuron	synaptic vesicle exocytosis	31
GO:0016202	muscle	regulation of striated muscle tissue development	71
GO:0016286	signalling	small conductance calcium-activated potassium channel activity	26
GO:0016301	signalling	kinase activity	144, 26
GO:0016307	signalling	phosphatidylinositol phosphate kinase activity	14, 2
GO:0016310	signalling	phosphorylation	144, 170, 26
GO:0016311	signalling	dephosphorylation	3
GO:0016319	neuron	mushroom body development	114, 52
GO:0016337	signalling	cell-cell adhesion	3
GO:0016360	morphology	sensory organ precursor cell fate determination	37
GO:0016477	morphology	cell migration	46
GO:0016567	signalling	protein ubiquitination	2
GO:0016568	morphology	chromatin modification	3
GO:0016746	signalling	transferase activity, transferring acyl groups	251
GO:0016757	signalling	transferase activity, transferring glycosyl groups	2, 3
GO:0016772	signalling	transferase activity, transferring phosphorus-containing groups	144
GO:0016791	signalling	phosphatase activity	3
GO:0016798	signalling	hydrolase activity, acting on glycosyl bonds	27
GO:0016820	signalling	hydrolase activity, acting on acid anhydrides, catalyzing transmembrane movement of substances	3
GO:0016887	regulation	ATPase activity	3
GO:0017017	signalling	MAP kinase tyrosine/serine/threonine phosphatase activity	3
GO:0017053	signalling	transcriptional repressor complex	37
GO:0017127	signalling	cholesterol transporter activity	74
GO:0018149	signalling	peptide cross-linking	3
GO:0018990	morphology	ecdysis, chitin-based cuticle	114
GO:0018996	morphology	molting cycle, collagen and cuticulin-based cuticle	114, 131, 46, 82, 92
GO:0019730	signalling	antimicrobial humoral response	114
GO:0019888	signalling	protein phosphatase regulator activity	267
GO:0019895	regulation	kinesin-associated mitochondrial adaptor activity	54
GO:0019896	neuron	axon transport of mitochondrion	54
GO:0019915	signalling	lipid storage	179, 19, 20, 22, 51, 5, 74
GO:0021884	neuron	forebrain neuron development	82
GO:0021905	neuron	forebrain-midbrain boundary formation	3
GO:0022008	neuron	neurogenesis	171, 267, 279, 46, 62
GO:0030001	signalling	metal ion transport	18
GO:0030097	signalling	hemopoiesis	18

. . . Table S4 continued

GO-ID	classification	GO description	internal COP-ID (ake-###)
GO:0030111	signalling	regulation of Wnt receptor signaling pathway	400, 60
GO:0030154	morphology	cell differentiation	71
GO:0030163	signalling	protein catabolic process	92
GO:0030239	muscle	myofibril assembly	16, 5, 82
GO:0030240	muscle	skeletal muscle thin filament assembly	203, 5
GO:0030289	signalling	protein phosphatase 4 complex	267
GO:0030301	signalling	cholesterol transport	74
GO:0030307	signalling	positive regulation of cell growth	32
GO:0030382	regulation	sperm mitochondrion organization	54
GO:0030414	signalling	peptidase inhibitor activity	162
GO:0030421	signalling	defecation	16
GO:0030707	signalling	ovarian follicle cell development	32
GO:0030916	signalling	otic vesicle formation	174
GO:0031016	morphology	pancreas development	60
GO:0031453	signalling	positive regulation of heterochromatin assembly	155
GO:0031618	signalling	nuclear centromeric heterochromatin	155
GO:0032012	signalling	regulation of ARF protein signal transduction	3
GO:0032474	morphology	otolith morphogenesis	174
GO:0034220	signalling	ion transmembrane transport	135, 3, 52
GO:0034605	signalling	cellular response to heat	155
GO:0034707	signalling	chloride channel complex	52
GO:0035023	signalling	regulation of Rho protein signal transduction	63
GO:0035050	morphology	embryonic heart tube development	3
GO:0035071	signalling	salivary gland cell autophagic cell death	323, 32
GO:0035072	signalling	ecdysone-mediated induction of salivary gland cell autophagic cell death	114
GO:0035098	signalling	ESC/E(Z) complex	3
GO:0035147	signalling	branch fusion, open tracheal system	101
GO:0035188	morphology	hatching	267
GO:0035199	signalling	salt aversion	3
GO:0035238	signalling	vitamin A biosynthetic process	64
GO:0035282	morphology	segmentation	32
GO:0035304	signalling	regulation of protein dephosphorylation	267
GO:0035307	signalling	positive regulation of protein dephosphorylation	142
GO:0035335	signalling	peptidyl-tyrosine dephosphorylation	3
GO:0035462	morphology	determination of left/right asymmetry in diencephalon	3
GO:0035469	morphology	determination of pancreatic left/right asymmetry	3
GO:0035556	signalling	intracellular signal transduction	184, 63
GO:0036065	signalling	fucosylation	2
GO:0040002	morphology	collagen and cuticulin-based cuticle development	18
GO:0040010	signalling	positive regulation of growth rate	102, 131, 150, 16, 171, 174, 203, 216, 232, 242, 255, 259, 2, 279, 46, 71, 82, 84, 92, 97
GO:0040017	signalling	positive regulation of locomotion	46
GO:0040018	morphology	positive regulation of multicellular organism growth	114, 174, 19
GO:0040020	morphology	regulation of meiosis	276
GO:0040026	morphology	positive regulation of vulval development	180
GO:0040034	morphology	regulation of development, heterochronic	114
GO:0040035	morphology	hermaphrodite genitalia development	120, 16, 171, 92
GO:0042048	neuron	olfactory behavior	55
GO:0042051	neuron	compound eye photoreceptor development	46
GO:0042063	neuron	gliogenesis	37
GO:0042074	morphology	cell migration involved in gastrulation	60
GO:0042127	morphology	regulation of cell proliferation	276
GO:0042332	signalling	gravitaxis	6
GO:0042673	neuron	regulation of retinal cone cell fate specification	82
GO:0042676	neuron	compound eye cone cell fate commitment	82
GO:0042745	morphology	circadian sleep/wake cycle	6
GO:0042749	morphology	regulation of circadian sleep/wake cycle	6

GO-ID	classification	GO description	internal COP-ID (ake-###)
GO:0042803	signalling	protein homodimerization activity	155, 32, 37
GO:0042981	signalling	regulation of apoptotic process	134
GO:0043005	neuron	neuron projection	6
GO:0043053	signalling	dauer entry	113
GO:0043066	signalling	negative regulation of apoptotic process	32
GO:0043086	signalling	negative regulation of catalytic activity	153
GO:0043152	signalling	induction of bacterial agglutination	2
GO:0043282	muscle	pharyngeal muscle development	71
GO:0043401	signalling	steroid hormone mediated signaling pathway	114
GO:0043524	neuron	negative regulation of neuron apoptotic process	51
GO:0043620	regulation	regulation of DNA-dependent transcription in response to stress	60
GO:0045087	signalling	innate immune response	233
GO:0045165	morphology	cell fate commitment	37
GO:0045179	signalling	apical cortex	82
GO:0045214	signalling	sarcomere organization	16, 5
GO:0045468	neuron	regulation of R8 cell spacing in compound eye	37
GO:0045475	neuron	locomotor rhythm	6
GO:0045500	neuron	sevenless signaling pathway	46
GO:0045572	signalling	positive regulation of imaginal disc growth	102
GO:0045664	neuron	regulation of neuron differentiation	82
GO:0045676	neuron	regulation of R7 cell differentiation	82
GO:0045746	signalling	negative regulation of Notch signaling pathway	32
GO:0045793	signalling	positive regulation of cell size	120
GO:0045892	regulation	negative regulation of transcription, DNA-dependent	37
GO:0045893	regulation	positive regulation of transcription, DNA-dependent	102
GO:0045900	regulation	negative regulation of translational elongation	285
GO:0045944	signalling	positive regulation of transcription from RNA polymerase II promoter	101, 114, 155
GO:0045980	signalling	negative regulation of nucleotide metabolic process	153
GO:0046008	signalling	regulation of female receptivity, post-mating	2
GO:0046331	signalling	lateral inhibition	18
GO:0046488	signalling	phosphatidylinositol metabolic process	14, 2
GO:0046692	signalling	sperm competition	2
GO:0046716	muscle	muscle cell homeostasis	16, 5
GO:0046843	morphology	dorsal appendage formation	32
GO:0046854	signalling	phosphatidylinositol phosphorylation	14, 2
GO:0046928	neuron	regulation of neurotransmitter secretion	31
GO:0046982	signalling	protein heterodimerization activity	101, 37
GO:0046983	signalling	protein dimerization activity	2
GO:0047291	signalling	lactosylceramide alpha-2, 3-sialyltransferase activity	3
GO:0048102	signalling	autophagic cell death	323, 32
GO:0048190	morphology	wing disc dorsal/ventral pattern formation	323, 37
GO:0048311	regulation	mitochondrion distribution	54
GO:0048368	morphology	lateral mesoderm development	3
GO:0048477	morphology	oogenesis	114, 32
GO:0048488	neuron	synaptic vesicle endocytosis	134, 46
GO:0048500	signalling	signal recognition particle	285
GO:0048512	morphology	circadian behavior	6
GO:0048514	morphology	blood vessel morphogenesis	63
GO:0048644	muscle	muscle organ morphogenesis	5
GO:0048666	neuron	neuron development	37
GO:0048676	neuron	axon extension involved in development	203
GO:0048738	muscle	cardiac muscle tissue development	16
GO:0048741	muscle	skeletal muscle fiber development	71
GO:0048749	neuron	compound eye development	32, 37, 51
GO:0048752	morphology	semicircular canal morphogenesis	174
GO:0048755	neuron	branching morphogenesis of a nerve	3

. . . Table S4 continued

GO-ID	classification	GO description	internal COP-ID (ake-###)
GO:0048812	neuron	neuron projection morphogenesis	3
GO:0048813	neuron	dendrite morphogenesis	37, 51, 82
GO:0048844	morphology	artery morphogenesis	6
GO:0048846	neuron	axon extension involved in axon guidance	14
GO:0048854	neuron	brain morphogenesis	37, 55
GO:0048885	neuron	neuromast deposition	82
GO:0048886	neuron	neuromast hair cell differentiation	82
GO:0048920	neuron	posterior lateral line neuromast primordium migration	162
GO:0050768	neuron	negative regulation of neurogenesis	37
GO:0050771	signalling	negative regulation of axonogenesis	82
GO:0050790	signalling	regulation of catalytic activity	3, 63
GO:0050830	signalling	defense response to Gram-positive bacterium	153
GO:0050909	signalling	sensory perception of taste	82
GO:0050914	signalling	sensory perception of salty taste	3
GO:0051124	neuron	synaptic growth at neuromuscular junction	31
GO:0051216	morphology	cartilage development	174, 3
GO:0051260	signalling	protein homooligomerization	3
GO:0051403	signalling	stress-activated MAPK cascade	155
GO:0051608	signalling	histamine transport	64
GO:0055002	muscle	striated muscle cell development	71
GO:0055059	neuron	asymmetric neuroblast division	82
GO:0055060	neuron	asymmetric neuroblast division resulting in ganglion mother cell formation	82
GO:0060028	neuron	convergent extension involved in axis elongation	400, 60
GO:0060029	morphology	convergent extension involved in organogenesis	60
GO:0060037	morphology	pharyngeal system development	174
GO:0060041	neuron	retina development in camera-type eye	64
GO:0060052	neuron	neurofilament cytoskeleton organization	51
GO:0060385	neuron	axonogenesis involved in innervation	82
GO:0060438	morphology	trachea development	174
GO:0060857	neuron	establishment of glial blood-brain barrier	152
GO:0060971	morphology	embryonic heart tube left/right pattern formation	3
GO:0061327	morphology	anterior Malpighian tubule development	174
GO:0070278	signalling	extracellular matrix constituent secretion	174
GO:0070527	signalling	platelet aggregation	3
GO:0070829	signalling	heterochromatin maintenance	155
GO:0070983	neuron	dendrite guidance	82
GO:0071436	signalling	sodium ion export	24, 3
GO:0071470	signalling	cellular response to osmotic stress	155
GO:0071688	muscle	striated muscle myosin thick filament assembly	203, 5
GO:0071805	signalling	potassium ion transmembrane transport	144, 26
GO:0072320	signalling	volume-sensitive chloride channel activity	19
GO:0072583	signalling	clathrin-mediated endocytosis	46
GO:0090305	signalling	nucleic acid phosphodiester bond hydrolysis	131
GO:2000223	signalling	regulation of BMP signaling pathway involved in heart jogging	3

Table S5. Supplementary Table related to Figures 1 and 5. Description of the 85 clusters in set L'.

internal ID	Flybase ID	Symbol	Name	Description	Ensembl ID	Symbol	Name	Description	Wormbase ID	Symbol	Name	Description	Supplementary Reference
	Fly				Fish				Worm				
**Morphology related**													
ake-114	FBgn0000448, FBgn0000568	Hr46, Eip75B	Hormone receptor-like in 46, Ecdysone-induced protein 75B	DHR3, a ecdysone-inducible gene product involved in metamorphosis	ENSDARG00000043059	VDR	vitamin D receptor		WBGene00003622	nhr-23	nuclear hormone receptor homolog	required in all larval molts for hypodermal expression and restricted to epidermal cells	[177, 113, 60, 112, 42]
ake-46	FBgn0000414	Dab	Disabled		ENSDARG00000053091, ENSDARG00000031761	Dab2, BDP1	disabled homolog 2, subunit of RNA polymerase III transcription initiation factor IIIB		WBGene00000894	dab-1	disabled ortholog	required for normal molting and meiotic arrest	[189, 65, 33, 63, 108, 156, 164]
ake-179	FBgn0264490	Eip93F	Ecdysone-induced protein 93F		ENSDARG00000070907	lcor	ligand dependent nuclear receptor corepressor		WBGene00011315	mbr-1			[13, 184, 56, 150]
ake-174	FBgn0026562	sparc	BM-40-SPARC		ENSDARG00000019353, ENSDARG00000074989	sparc, sparcl1	secreted acidic cysteine rich glycoprotein, SPARC-like 1		WBGene00003893	ost-1	ortholog of the conserved basement membrane glycoprotein component osteonectin/-SPARC/BM-40	required for embryonic and larval development	[97, 18, 180, 58, 27, 200, 135]
ake-14	FBgn0259244				ENSDARG00000037845, ENSDARG00000039462, ENSDARG00000040118, ENSDARG00000054753, ENSDARG00000058960, ENSDARG00000060893, ENSDARG00000068218, ENSDARG00000076163	Col9a3, col10a1, col8a2, otolin 1	collagen		WBGene00000708	col-135	collagen		[111, 45, 3, 109]
ake-101	FBgn0039411	dys	dysfusion		ENSDARG00000055752, ENSDARG00000061920, ENSDARG00000087753	npas4	neuronal PAS domain protein 4 (a, b)		WBGene00000521	cky-1	encodes a member of the basic helix-loop-helix/Per-Arnt-Sim (bHLH-PAS) protein family		[101, 102]
ake-63	FBgn0035308				ENSDARG00000040614, ENSDARG00000076480, ENSDARG00000040614	Sestd1, mcf2l	SEC14 and spectrin domains, mcf.2 cell line derived transforming sequence-like b		WBGene00010111, WBGene00018887, WBGene00194671				[144, 214]
ake-32	FBgn0259176	bun	bunched		ENSDARG00000038306, ENSDARG00000075666	Tsc22d (1, 3)	TSC22 domain family, member (1, 3)		WBGene00016272, WBGene00011824				[64, 69, 7, 107]
**Muscle related**													
ake-16	FBgn0004028	wupA	wings up A	troponin I (TnI)	ENSDARG00000005841, ENSDARG00000013752, ENSDARG00000029069, ENSDARG00000029995, ENSDARG00000035958, ENSDARG00000042559, ENSDARG00000045592, ENSDARG00000052708, ENSDARG00000073766	tnni (2a.2, 2a.3, 2a.4, 2b.2, 2b.1, 1c, 2a.1, 1b, 1d)	troponin I, skeletal, fast, tandem duplicate		WBGene00006584, WBGene00006585, WBGene00006586, WBGene00006764	Tni-(1,3,4), unc-76	troponin I	encodes a member of the troponin family that affects body morphology, locomotion, egg laying, and epithelial morphogenesis	[37, 190, 73, 80, 157, 137, 132, 154, 88, 61, 194, 152]

. . . Table S5 continued

internal ID	Flybase ID	Symbol	Name	Description	Ensembl ID	Symbol	Name	Description	Wormbase ID	Symbol	Name	Description	References in Supplementary
	Fly				Fish				Worm				
ake-5	FBgn0004169	up	upheld	troponin T (TnT)	ENSDARG00000002988, ENSDARG00000030270, ENSDARG00000037954, ENSDARG00000045822, ENSDARG00000068457	tnnt (2d, 3a, 2e, 3b)	Troponin T (2d, 2e -cardiac; 3a, 3b -skeletal; typ 1 -skeletal)		WBGene00006588	Tnt-3	troponin T	orthologous to the human gene TROPONIN T (TNNT2; OMIM:191045), which when mutated leads to disease	[37, 190, 73, 80, 157, 137, 132, 154, 88, 61, 194, 152]
ake-71	FBgn0002922	nau	nautilus		ENSDARG00000007277, ENSDARG00000029830, ENSDARG00000030110	Myf (5, 6, d1)	myogenic factor (5, 6, d1)		WBGene00001948	hlh-1	ortholog of the myogenic regulatory factor (MRF) subgroup of basic helix-loop-helix (bHLH) transcription factor	required during embryogenesis for proper bodywall muscle development and function, but not for bodywall muscle cell fate specification	[54, 8, 30, 12, 4, 143, 208, 125, 195, 170]
ake-33	FBgn0003016	osp	outspread		ENSDARG00000006385, ENSDARG00000073998, ENSDARG00000074705	Triobp, X	TRIO and F-actin binding protein, myosin phosphatase Rho interacting protein		WBGene00008666				[141]
ake-120	FBgn0035411	Girdin	Girdin		ENSDARG00000076189, ENSDARG00000078440	Ccdc88 (b, Aa)	coiled-coil domain containing (88B, 88Aa)		WBGene00013082				[162, 205, 159]
ake-188	FBgn0039869				ENSDARG00000016754	tbca	tubulin cofactor a		WBGene00021475				[103, 222]
ake-225	FBgn0037737	Pnn	Pinin		ENSDARG00000015851	pnn	pinin	desmosome associated protein	WBGene00011308				[204, 104, 210, 89, 90]
ake-169	FBgn0030699				ENSDARG00000010400	lrrfip2	leucine rich repeat (in FLII) interacting protein 2		WBGene00018998	flap-1			[43]
ake-113	FBgn0036196				ENSDARG00000040277, ENSDARG00000075172	Fbxo (32, 25)	F-box protein (32, 25)		WBGene00008439	mfb-1	F-box protein		[41, 99, 72]
ake-144	FBgn0030745				ENSDARG00000024047, ENSDARG00000041562	Tmem38a, X	transmembrane protein 38A, Uncharacterized protein		WBGene00013255, WBGene00013268				[212]
ake-68	FBgn0035772	Sh3beta	SH3-binding, glutamic acid-rich protein		ENSDARG00000021633, ENSDARG00000036878, ENSDARG00000058302	sh3bgr, sh3bgrl2	SH3 domain binding glutamic acid-rich protein, like		WBGene00013666				[140]
							**Neuron related**						
ake-267	FBgn0030208	PPP4R2r	Protein phosphatase 4 regulatory subunit 2-related protein		ENSDARG00000026540	ppp4r2a	protein phosphatase 4 regulatory subunit 2a		WBGene00017064	Ppfr-2			[17]
ake-251	FBgn0015323	VAChT			ENSDARG00000006356, ENSDARG00000090189	Slc18a3a, X	solute carrier family 18 (vesicular acetylcholine) member 3a, Uncharacterized protein		WBGene00000481, WBGene00006756	Cha-1, unc-17	Cha-1 encodes a choline acetyltransferase that synthesizes acetylcholine, unc-17 encodes a synaptic vesicle acetylcholine transporter (VAChT), share a common promoter	expressed in neurons, and is required for viability, normal growth, locomotion, and sensitivity to acetylcholinesterase inhibitors	[44, 83, 51]

... Table S5 continued

internal ID	Flybase ID	Symbol	Name	Description	Ensembl ID	Symbol	Name	Description	Wormbase ID	Symbol	Name	Description	References in Supplementary
	Fly				Fish				Worm				
ake-25	FBgn0051057	tau			ENSDARG00000055052, ENSDARG00000087616, ENSDARG00000089314	Mapt (a, b), tau a	microtubule-associated protein (a, b), microtubule-associated protein tau		WBGene00004212	ptl-1	microtubule-binding protein homologous to members of the tau/MAP2/MAP4 subfamily of microtubule-associated proteins	associated with several neurodegenerative disorders	[31, 55, 98, 16, 62, 114]
ake-82	FBgn0004595	pros	prospero		ENSDARG00000041952, ENSDARG00000055158	Prox (2, 1a)	prospero homeobox (2, 1a)		WBGene00000448	Ceh-26	encodes a protein that contains a prospero-related homeodomain		[46, 161, 6, 219]
ake-276	FBgn0039977				ENSDARG00000056235	smndc1	survival motor neuron domain containing 1		WBGene00004891	smr-1			[191]
ake-31	FBgn0041605	cpx	complexin		ENSDARG00000018997, ENSDARG00000061918	cplx2	Complexin 2, like		WBGene00022271	cpx-1			[142, 70, 133, 81, 34, 95, 218]
ake-64	FBgn0260964	Vmat	Vesicular monoamine transporter		ENSDARG00000015110, ENSDARG00000078157	Slc18a2, X	solute carrier family 18 (vesicular monoamine) member 2, solute carrier family 18 (vesicular monoamine transporter) member 1		WBGene00000295	cat-1	encodes a synaptic vesicular monoamine transporter	required for the presence of dopamine and serotonin in nerve terminals	[50]
ake-62	FBgn0040395	Unc-76			ENSDARG00000022260, ENSDARG00000023174, ENSDARG00000073784		fasciculation and elongation protein zeta (1, 2)		WBGene00006808	unc-76	coiled-coil protein that belongs to the FEZ (fasciculation and elongation protein; zygin/zeta-1) family of proteins	required for normal axonal outgrowth and fasciculation and hence, normal locomotion and is present in all axons throughout development	[119, 199, 10, 67, 38, 15, 139]
ake-37	FBgn0000591, FBgn0002631, FBgn0002633, FBgn0002735, FBgn0032741	E(spl), HLHm5, HLHm7, HLHm-beta, HLH-mgamma, Side	Enhancer of split, E(spl) region transcript m (5, 7, mbeta, mgamma), similar to Deadpan	Enhancer of split/region transcript m5/.../similar to Deadpan	ENSDARG00000007097, ENSDARG00000016363, ENSDARG00000019335, ENSDARG00000068168, ENSDARG00000069675	her (13, 8a, 2.2, 8.2), hes (6, 2.2)	Hairy-related (13, 8a, 8.2), hairy-related 8a, hairy and enhancer of split 2		WBGene00003008				[207, 9, 66, 115, 220, 57, 181, 39, 85]
ake-73	FBgn0032949, FBgn0052225	Lamp1, X			ENSDARG00000077944, ENSDARG00000092610		lysosomal-associated membrane protein 1		WBGene00015471	lmp-2	lysosomal associated membrane glycoprotein transmembrane protein		[68, 59]
ake-21	FBgn0031213, FBgn0034365	Galectin, X			ENSDARG00000092923, ENSDARG00000093059, ENSDARG00000037613, ENSDARG00000044001, ENSDARG00000077850	Galectin, lectin, lgals3l	Galactoside-binding		WBGene00002266, WBGene00002272, WBGene00008284, WBGene00017080, WBGene00002271, WBGene00002270, WBGene00002273, WBGene00002268	Lec-9,8,7,10,5,3			[87]
ake-155	FBgn0050420	Atf-2	Activating transcription factor-2		ENSDARG00000056156, ENSDARG00000074697	Npdc1, X	neural proliferation, differentiation and control 1		WBGene00000277	cab-1		involved in synaptic regulation	[1]

. . . Table S5 continued

internal ID	Flybase ID	Symbol	Name	Description	Ensembl ID	Symbol	Name	Description	Wormbase ID	Symbol	Name	Description	References in Supplementary
	Fly				Fish				Worm				
ake-6	FBgn0030437, FBgn0033932, FBgn0052843, FBgn0260753	Hector, Dh44-R1, Dh31-R1, Pdfr	Hector, Diuretic hormone (44, 31) receptor 1, Pigment-dispersing factor receptor	Diuretic hormone 31 receptor /Pigment-dispersing factor receptor	ENSDARG00000003989, ENSDARG00000006678, ENSDARG00000011473, ENSDARG00000011571, ENSDARG00000018418, ENSDARG00000020957, ENSDARG00000021186, ENSDARG00000028845, ENSDARG00000062377	Crhr1, pth2r, calcrla, calcrlb, pth1rb, pth1ra, calcr	corticotropin, parathyroid, calcitonin hormone receptor		WBGene00007664, WBGene00014035		G-protein-coupled receptor (GPCR) that is a member of the secretin family		[44, 197, 117, 25, 100, 145]
ake-203	FBgn0036641	Smn	survival motor neuron		ENSDARG00000018494	smn1	survival motor neuron 1		WBGene00004887	smn-1	encodes a homolog of human SMN	related to muscle	[53]
ake-51	FBgn0259108	futsch			ENSDARG00000022045, ENSDARG00000059601, ENSDARG00000060326, ENSDARG00000060434, ENSDARG00000060805	map	microtubule-associated protein (1Ab, 1Aa, 1S, 1b)		WBGene00007966, WBGene00009113, WBGene00009306				[79, 94, 175]
ake-26	FBgn0029761,	SK	small conductance calcium-activated potassium channel		ENSDARG00000014939, ENSDARG00000019753, ENSDARG00000023546, ENSDARG00000091306	Kcnn (1a, 1b, 3)	potassium intermediate/small conductance calcium-activated channel subfamily N member (2, 3, 1b, 1a)		WBGene00008570	Kcnl-2			[47]

. . . Table S5 continued

internal ID	Flybase ID	Symbol	Name	Description	Ensembl ID	Symbol	Name	Description	Wormbase ID	Symbol	Name	Description	References in Supplementary
	Fly				Fish				Worm				
ake-3	FBgn0034286, FBgn0034723, FBgn0037993, FBgn0040726, FBgn0040823, FBgn0051361, FBgn0052600, FBgn0053202, FBgn0053481, FBgn0085400	Dpr (13, 15, 6, 17, 8, 11, 7)		defective proboscis extension response	ENSDARG00000015607, ENSDARG00000017984, ENSDARG00000028912, ENSDARG00000029838, ENSDARG00000034092, ENSDARG00000034466, ENSDARG00000040669, ENSDARG00000041828, ENSDARG00000043445, ENSDARG00000045875, ENSDARG00000053027, ENSDARG00000054655, ENSDARG00000055245, ENSDARG00000057013, ENSDARG00000057886, ENSDARG00000058564, ENSDARG00000070065, ENSDARG00000070223, ENSDARG00000071050, ENSDARG00000076144, ENSDARG00000076981, ENSDARG00000078180, ENSDARG00000078824, ENSDARG00000079487, ENSDARG00000086166, ENSDARG00000086301, ENSDARG00000086919, ENSDARG00000087148, ENSDARG00000088811, ENSDARG00000089316, ENSDARG00000089404, ENSDARG00000089710, ENSDARG00000089993, ENSDARG00000090329, ENSDARG00000090874, ENSDARG00000091074, ENSDARG00000091388, ENSDARG00000091579, ENSDARG00000091661, ENSDARG00000091712, ENSDARG00000092031, ENSDARG00000092322, ENSDARG00000092520, ENSDARG00000093279, ENSDARG00000093880, ENSDARG00000093987, ENSDARG00000094374, ENSDARG00000094391, ENSDARG00000094524, ENSDARG00000095133, ENSDARG00000095134, ENSDARG00000095772	cadm3, unknown	cell adhesion molecule 3, Uncharacterized proteins		WBGene00007851, WBGene00021117, WBGene00021290, WBGene00021291, WBGene00006985	Clec-(245, 126, 122, 121), zig-8	predicted secreted proteins	member of the immunoglobulin superfamily	[155, 71]
ake-162	FBgn0039155	kal-1	Kallmann syndrome 1 ortholog		ENSDARG00000004932, ENSDARG00000012896, ENSDARG00000087064	Kal1 (b, a)	Kallmann syndrome 1 (b, a) sequence		WBGene00002181	kal-1	cell surface protein that contains a WAP-type protease inhibitor domain and Type III fibronectin domains	ortholog of human KAL-1, required for epithelial morphogenesis and axon branching	[178, 202]
ake-84	FBgn0037443				ENSDARG00000046007, ENSDARG00000060954	Tmcc2, X	transmembrane and coiled-coil domain family (2, 1b)		WBGene00015800				[221, 84]

...Table S5 continued

internal ID	Flybase ID	Symbol	Name	Description	Ensembl ID	Symbol	Name	Description	Wormbase ID	Symbol	Name	Description	References in Supplementary
	Fly				Fish				Worm				
ake-24	FBgn0030638, FBgn0030756				ENSDARG00000009131, ENSDARG00000030588, ENSDARG00000095660	Slc10a (4, 3, 1)	solute carrier family 10 (sodium/bile acid cotransporter family) member (4, 3, 1)		WBGene00022135				[120, 167]
ake-112	FBgn0035103, FBgn0042101	Vdup1, X	Vitamin D[[3]] up-regulated protein 1		ENSDARG00000032444, ENSDARG00000067664		Uncharacterized proteins		WBGene00013043	arrd-16			[123, 28]
ake-224	FBgn0032221				ENSDARG00000035175	schip1	schwannomin interacting protein 1		WBGene00018074				[74, 134]
ake-334	FBgn0035676				ENSDARG00000000588	grasp	GRP1 (general receptor for phosphoinositides 1)-associated scaffold protein		WBGene00009272				[110]
**Signalling related**													
ake-74	FBgn0035028	start1			ENSDARG00000017809, ENSDARG00000074280	Stard3, X	START domain containing 3, StAR-related lipid transfer (START) domain containing 4		WBGene00017826, WBGene0001050				[11, 176]
ake-134	FBgn0261722	fwe	flower		ENSDARG00000090818		calcium channel flower domain containing 1		WBGene00008973				[173, 168, 122, 129]
ake-152	FBgn0039014				ENSDARG00000057322	tmem47	transmemebrane protein 47		WBGene00006875	vab-9	claudin homolog orthologous to human brain cell membrane protein 1 (BCMP1)	Claudins are integral membrane proteins with four transmembrane sequences	[185, 91, 186]
ake-52	FBgn0015558, FBgn0261444	tty	tweety		ENSDARG00000007678, ENSDARG00000010727, ENSDARG00000016934, ENSDARG00000034473	Ttyh (3b, 2l, 2 3a)	tweety homolog (3b, 2, 2 like, 3a)		WBGene00009632	ttyh-1			[23, 138]
ake-18	FBgn0029506, FBgn0033124, FBgn0033127, FBgn0033128, FBgn0033129, FBgn0033130, FBgn0033133, FBgn0033134, FBgn0033135, FBgn0043550	Tsp42E (e, c, f, g, h, i, k, l, n), Tsp68C	Tetraspanin 42E (e, c, f, g, h, i, k, l, n), Tetraspanin 68C		ENSDARG00000015015, ENSDARG00000025757, ENSDARG00000068233	Tspan18b, X, X	tetraspanin 18b, X, CD53 molecule		WBGene00006637, WBGene00006643, WBGene00006635, WBGene00006642, WBGene00006641, WBGene00006639	Tsp-(11, 17, 9, 16, 15, 13)			[93, 149, 29, 148]

. . . Table S5 continued

internal ID	Flybase ID	Symbol	Name	Description	Ensembl ID	Symbol	Name	Description	Wormbase ID	Symbol	Name	Description	References in Supplementary
	Fly				Fish				Worm				
ake-19	FBgn0035696, FBgn0040238	Best (2, 1)	Bestrophin (2, 1)	Bestrophin 2/1	ENSDARG00000078331, ENSDARG00000078455, ENSDARG00000079163	Best (1, 4, 2)	Bestrophin (1, 4, 2)		WBGene00015288, WBGene00007404, WBGene00015628, WBGene00007808, WBGene00007988, WBGene00008185, WBGene00008186, WBGene00008821, WBGene00011258, WBGene00020559, WBGene00011868, WBGene00021368, WBGene00013520, WBGene00013921, WBGene00022797, WBGene00014102, WBGene00014103		orthologous to the human gene VITELLIFORM MACULAR DYSTROPHY PROTEIN (VMD2)		[35, 36, 193]
ake-22	FBgn0037913	fabp	fatty acid bindin protein		ENSDARG00000007697, ENSDARG00000023290, ENSDARG00000034650, ENSDARG00000035904, ENSDARG00000045926	Fabp(7a, 3, 7b) crabp1 (b, a)	fatty acid binding protein (7 – brain, 3 - muscle), cellular retinoic acid binding protein 1 (a, b)		WBGene00002259, WBGene00002260, WBGene00002258, WBGene00002257, WBGene00021486	Lbp-(7, 8, 6, 5, 9)	intracellular fatty acid binding protein (iFABP)	predicted to function as an intracellular transporter for small hydrophobic molecules such as lipids and steroid hormones	[2, 151, 147]
ake-102	FBgn0036318		Wbp2 ortholog		ENSDARG00000056605	Wbp2	WW domain binding protein 2		WBGene00015955, WBGene00008404				[223]
ake-180	FBgn0021818	cnk	connector enhancer of ksr		ENSDARG00000074236				WBGene00000564	cnk-1	protein that contains a SAM domain, a PDZ domain, and a PH domain		[40, 20, 198, 174]
ake-285	FBgn0038808	Srp14	Signal recognition particle protein 14		ENSDARG00000088272	srp14	signal recognition particle 14		WBGene00017799				[49, 14]
ake-401	FBgn0030711				ENSDARG00000021112	c1d	C1D nuclear receptor co-repressor		WBGene00021785				[215]
ake-55	FBgn0032156, FBgn0034181				ENSDARG00000042892	paip1	poly(A) binding protein interacting protein 1		WBGene00018405				[130]
ake-60	FBgn0033166	Eaf	ELL-associated factor		ENSDARG00000010432, ENSDARG00000070795	Eaf (2, 1)	ELL associated factor (2, 1)		WBGene00017011	eaf-1			[187, 126, 21, 22, 19, 118, 169, 131, 127]
ake-400	FBgn0035148				ENSDARG00000059177	tax1bp3	Tax1 (human T-cell leukemia virus type I) binding protein 3		WBGene00016678				[171]
ake-20	FBgn0034345				ENSDARG00000013655, ENSDARG00000027154, ENSDARG00000042548	Tpd52l (1, 2b, 2a)	tumor protein D52-like (2b, 2a, 1)		WBGene00008745				[24]
ake-92	FBgn0037299				ENSDARG00000028391, ENSDARG00000031836, ENSDARG00000039319	vps37	vacuolar protein sorting 37 homolog (B, C)		WBGene00016990	vps-37	endosomal sorting ESCRT-I complex that functions in endosomal sorting of mono-ubiquitinated membrane proteins		[160, 76]
ake-233	FBgn0036847				ENSDARG00000070512	tnrc5	trinucleotide repeat containing 5		WBGene00007531				[203]

. . . Table S5 continued

internal ID	Flybase ID	Symbol	Name	Description	Ensembl ID	Symbol	Name	Description	Wormbase ID	Symbol	Name	Description	References in Supplementary
	Fly				Fish				Worm				
ake-142	FBgn0036090				ENSDARG00000036064	Cnep1r1	CTD nuclear envelope phosphatase 1 regulatory subunit 1		WBGene00011828				[77]
							**Regulation related**						
ake-54	FBgn0262872	milt	milton		ENSDARG00000041304, ENSDARG00000078872	trak2	trafficking protein kinesin binding (2, 1)		WBGene00020838				[98, 121, 201]
ake-11	FBgn0034067, FBgn0039648, FBgn0261534		l(2)34Fc lethal (2) 34Fc		ENSDARG00000060622, ENSDARG00000069103, ENSDARG00000093957		ferric-chelate reductase 1 like		WBGene00007339, WBGene00007545, WBGene00013292				[166]
ake-118	FBgn0011455		l(3)neo18 lethal (3) neo18		ENSDARG00000070824	ndufb5	NADH dehydrogenase (ubiquinone) 1 beta subcomplex 5		WBGene00016118	ortholog of the NDUFB5/SGDH subunit of the mitochondrial NADH dehydrogenase (ubiquinone) complex (complex I)			[192, 163]
ake-131	FBgn0262116				ENSDARG00000069336, ENSDARG00000069336, ENSDARG00000069461	Rnasek (b, a)	ribonuclease, RNase K (b, a)		WBGene00016721, WBGene00009881				[106]
ake-135	FBgn0032833, FBgn0033020		Cytochrome c oxidase subunit IV		ENSDARG00000022509, ENSDARG00000032970	Cox4i (2, 1)	Cytochrome c oxidase subunit IV isoform (2, 1)		WBGene00012354				[146, 92, 183, 5, 165]
ake-150	FBgn0260407	mRpS23	mitochondrial Ribosomal protein S23		ENSDARG00000033973	Mrps23	mitochondrial ribosomal protein S23		WBGene00014224				[179, 158, 26, 136, 188]
ake-153	FBgn0040660				ENSDARG00000044092, ENSDARG00000067975	Atpif1 (b, a)	ATPase inhibitory factor 1 (b, a)		WBGene00015248	mai-2	orthologous to mitochondrial intrinsic ATPase inhibitor protein IF(1))	blocks reverse action (ATP hydrolysis) by F(0)F(1)-ATPase when its (normally required) proton gradient is lost	[32, 96, 182]
ake-170	FBgn0033373				ENSDARG00000007181	nadk2	NAD kinase 2 mitochondrial		WBGene00012463				[86]
ake-171	FBgn0053051				ENSDARG00000012044, ENSDARG00000024687	polr3gla, polr3g	polymerase (RNA) III (DNA directed) polypeptide G, like a		WBGene00021638				[206, 172, 209]
ake-216	FBgn0260460	mRpS34	mitochondrial ribosomal protein S34		ENSDARG00000057910	mrps34	mitochondrial ribosomal protein S34		WBGene00010905				[179, 158, 26, 136, 188]
ake-232	FBgn0037566	mRpL1	mitochondrial ribosomal protein L1		ENSDARG00000054606	mrpl1	mitochondrial ribosomal protein L1		WBGene00017997				[179, 158, 26, 136, 188]
ake-242	FBgn0031357	mRpL48	mitochondrial ribosomal protein L48		ENSDARG00000035167	mrpl48	mitochondrial ribosomal protein L48		WBGene00016989				[179, 158, 26, 136, 188]
ake-255	FBgn0038426	mRpS33	mitochondrial ribosomal protein S33		ENSDARG00000020015	mrps33	mitochondrial ribosomal protein S33		WBGene00009013				[179, 158, 26, 136, 188]
ake-259	FBgn0032053	mRpL51	mitochondrial ribosomal protein L51		ENSDARG00000041340		mitochondrial ribosomal protein L51		WBGene00011740				[179, 158, 26, 136, 188]

. . . Table S5 continued

internal ID	Flybase ID	Symbol	Name	Description	Ensembl ID	Symbol	Name	Description	Wormbase ID	Symbol	Name	Description	References in Supplementary
	Fly				Fish				Worm				
ake-97	FBgn0034986	mRpS17	mitochondrial ribosomal protein S17		ENSDARG00000054164		mitochondrial ribosomal protein S17		WBGene00015487				[179, 158, 26, 136, 188]
ake-327	FBgn006411				ENSDARG00000056855		glutamyl-tRNA(Gln) amidotransferase subunit C		WBGene00013433				[52, 153]
ake-160	FBgn0038400				ENSDARG00000039374		apolipoprotein O-like		WBGene00019333	moma-1			[78]
ake-217	FBgn0025820	JTBR			ENSDARG00000070150		jumping translocation breakpoint		WBGene00002180	jtr-1	predicted transmembrane protein that is related to the conserved jumping translocation breakpoint (JTB) family of proteins		[105]
ake-323	FBgn0037700				ENSDARG00000059357	sarnp	SAP domain containing ribonucleoprotein		WBGene00021813				[116, 213]
ake-193	FBgn0032200				ENSDARG00000040822, ENSDARG00000086461	Fundc (1, 2)	FUN14 domain containing (1, 2)		WBGene00011528				[211, 128]

Others

internal ID	Flybase ID	Symbol	Name	Description	Ensembl ID	Symbol	Name	Description	Wormbase ID	Symbol	Name	Description	References in Supplementary
ake-184	FBgn0031413				ENSDARG00000086655, ENSDARG00000089374		chromosome 3 open reading frame 83		WBGene00021131				
ake-185	FBgn0032488				ENSDARG00000020611		chromosome 19 open reading frame 47		WBGene00010002				

...Table S5 continued

internal ID	Flybase ID	Symbol	Name	Description	Ensembl ID	Symbol	Name	Description	Wormbase ID	Symbol	Name	Description	References in Supplementary
	Fly				Fish				Worm				
ake-2	FBgn0016675, FBgn0031273, FBgn0031910, FBgn0040093, FBgn0040097, FBgn0040099, FBgn0040102, FBgn0040104, FBgn0040106, FBgn0040107, FBgn0053532, FBgn0053533, FBgn0259230, FBgn0259958, FBgn0262357	Lectin-(galC1, 46Ca, 30A, 28C, 24Db, 24A, 21Cb, 21Ca, 27Da, 37Db, 22C), Sfp24F	Galactose-specific C-type lectin, lectin-(46Ca, 30A, 28C, 24Db, 24A, 21Cb, 21Ca, 27Da, 37Db, 22C), Seminal fluid protein 24F		ENSDARG00000014624, ENSDARG00000021678, ENSDARG00000027399, ENSDARG00000034330, ENSDARG00000036745, ENSDARG00000038551, ENSDARG00000041159, ENSDARG00000041389, ENSDARG00000043736, ENSDARG00000043768, ENSDARG00000043769, ENSDARG00000045867, ENSDARG00000053113, ENSDARG00000058023, ENSDARG00000067495, ENSDARG00000070414, ENSDARG00000070850, ENSDARG00000070851, ENSDARG00000075003, ENSDARG0000007503, ENSDARG00000075634, ENSDARG00000076541, ENSDARG00000077136, ENSDARG00000077692, ENSDARG00000078750, ENSDARG00000079039, ENSDARG00000079107, ENSDARG00000086100, ENSDARG00000087115, ENSDARG00000088252, ENSDARG00000088614, ENSDARG00000088919, ENSDARG00000089758, ENSDARG00000090092, ENSDARG00000091095, ENSDARG00000091460, ENSDARG00000092749, ENSDARG00000092837, ENSDARG00000093391, ENSDARG00000093673, ENSDARG00000093909, ENSDARG00000094184, ENSDARG00000094225, ENSDARG00000094453, ENSDARG00000094750, ENSDARG00000095091, ENSDARG00000095335, ENSDARG00000095481, ENSDARG00000095757,	ly75, Uncharacterized proteins	lymphocyte antigen 75, Uncharacterized proteins		WBGene00015631, WBGene00009517, WBGene00009518, WBGene00018910, WBGene00044719, WBGene00021224, WBGene00021580, WBGene00021879, WBGene00021895, WBGene00023484, WBGene00022261, WBGene00014137, WBGene00014138	Clec-(89, 167, 166, 172, 209, 174, 83, 84, 210, 187, 186)			[196]
ake-279	FBgn0030061				ENSDARG00000013489	gltscr2	glioma tumor suppressor candidate region gene 2		WBGene00012692				[216, 217]
ake-27	FBgn0000078	Amy-d	Amylase distal		ENSDARG00000009443, ENSDARG00000030357				WBGene00008220				[82]

References for Supplementary Material

1. J. Ackermann, G. Ashton, S. Lyons, D. James, J.P. Hornung, N. Jones, and W. Breitwieser. Loss of ATF2 function leads to cranial motoneuron degeneration during embryonic mouse development. *PLoS One*, 6(4):e19090, 2011.

2. H.J. Ahn, S.H. Jeon, and S.H. Kim. Expression of a set of glial cell-specific markers in the Drosophila embryonic central nervous system. *BMB Rep*, 47(6):354–359, Jun 2014.

3. D. Aldea, P. Hanna, D. Munoz, J. Espinoza, M. Torrejon, L. Sachs, N. Buisine, S. Oulion, H. Escriva, and S. Marcellini. Evolution of the vertebrate bone matrix: an expression analysis of the network forming collagen paralogues in amphibian osteoblasts. *J Exp Zool B Mol Dev Evol*, 320(6):375–384, Sep 2013.

4. A.A. Amali, C.J. Lin, Y.H. Chen, W.L. Wang, H.Y. Gong, C.Y. Lee, Y.L. Ko, J.K. Lu, G.M. Her, T.T. Chen, and J.L. Wu. Up-regulation of muscle-specific transcription factors during embryonic somitogenesis of zebrafish (*Danio rerio*) by knock-down of myostatin-1. *Dev Dyn*, 229(4):847–856, Apr 2004.

5. S. Aras, O. Pak, N. Sommer, R. Finley, Jr., M. Huttemann, N. Weissmann, and L.I. Grossman. Oxygen-dependent expression of cytochrome c oxidase subunit 4-2 gene expression is mediated by transcription factors RBPJ, CXXC5 and CHCHD2. *Nucleic Acids Res*, 41(4):2255–2266, Feb 2013.

6. C.L. Araya, T. Kawli, A. Kundaje, L. Jiang, B. Wu, D. Vafeados, R. Terrell, P. Weissdepp, L. Gevirtzman, D. Mace, W. Niu, A.P. Boyle, D. Xie, L. Ma, J.I. Murray, V. Reinke, R.H. Waterston, and M. Snyder. Regulatory analysis of the *C. elegans* genome with spatiotemporal resolution. *Nature*, 512(7515):400–405, Aug 2014.

7. D.M. Ash, J.F. Hackney, M. Jean-Francois, N.C. Burton, and L.L. Dobens. A dominant negative allele of the Drosophila leucine zipper protein Bunched blocks bunched function during tissue patterning. *Mech Dev*, 124(7-8):559–569, Aug 2007.

8. A. Aziz, Q.C. Liu, and F.J. Dilworth. Regulating a master regulator: establishing tissue-specific gene expression in skeletal muscle. *Epigenetics*, 5(8):691–695, Nov 2010.

9. S. Bae, Y. Bessho, M. Hojo, and R. Kageyama. The bHLH gene Hes6, an inhibitor of Hes1, promotes neuronal differentiation. *Development*, 127(13):2933–2943, Jul 2000.

10. B.J. Barsi-Rhyne, K.M. Miller, C.T. Vargas, A.B. Thomas, J. Park, M. Bremer, J.L. Jarecki, and M.K. VanHoven. Kinesin-1 acts with netrin and DCC to maintain sensory neuron position in *Caenorhabditis elegans*. *Genetics*, 194(1):175–187, May 2013.

11. M.P. Bauer, J.T. Bridgham, D.M. Langenau, A.L. Johnson, and F.W. Goetz. Conservation of steroidogenic acute regulatory (StAR) protein structure and expression in vertebrates. *Mol Cell Endocrinol*, 168(1-2):119–125, Oct 2000.

12. L.R. Baugh and C.P. Hunter. Myod, modularity, and myogenesis: conservation of regulators and redundancy in *C. elegans*. *Genes Dev*, 20(24):3342–3346, Dec 2006.

13. M. Bender, F.B. Imam, W.S. Talbot, B. Ganetzky, and D.S. Hogness. Drosophila ecdysone receptor mutations reveal functional differences among receptor isoforms. *Cell*, 91(6):777–788, Dec 1997.

14. M. Blank and T. Burmester. Widespread occurrence of N-terminal acylation in animal globins and possible origin of respiratory globins from a membrane-bound ancestor. *Mol Biol Evol*, 29(11):3553–3561, Nov 2012.

15. L. Bloom and H.R. Horvitz. The *Caenorhabditis elegans* gene unc-76 and its human homologs define a new gene family involved in axonal outgrowth and fasciculation. *Proc Natl Acad Sci U S A*, 94(7):3414–3419, Apr 1997.

16. B.J. Bolkan and D. Kretzschmar. Loss of tau results in defects in photoreceptor development and progressive neuronal degeneration in Drosophila. *Dev Neurobiol*, 74(12):1210–1225, Dec 2014.

17. Y. Bosio, G. Berto, P. Camera, F. Bianchi, C. Ambrogio, P. Claus, and F. Di Cunto. PPP4R2 regulates neuronal cell differentiation and survival, functionally cooperating with SMN. *Eur J Cell Biol*, 91(8):662–674, Aug 2012.

18. A.D. Bradshaw and E.H. Sage. SPARC, a matricellular protein that functions in cellular differentiation and tissue response to injury. *J Clin Invest*, 107(9):1049–1054, May 2001.

19. D.J. Burgess. Gene expression: Time flies thanks to Pol II pausing. *Nat Rev Genet*, 14(7):441, Jul 2013.

20. C. Cabernard and M. Affolter. Distinct roles for two receptor tyrosine kinases in epithelial branching morphogenesis in Drosophila. *Dev Cell*, 9(6):831–842, Dec 2005.

21. L. Cai, B.L. Phong, A.L. Fisher, and Z. Wang. Regulation of fertility, survival, and cuticle collagen function by the *Caenorhabditis elegans* eaf-1 and ell-1 genes. *J Biol Chem*, 286(41):35915–35921, Oct 2011.

22. L. Cai, D. Wang, A.L. Fisher, and Z. Wang. Identification of a genetic interaction between the tumor suppressor EAF2 and the retinoblastoma protein (Rb) signaling pathway in *C. elegans* and prostate cancer cells. *Biochem Biophys Res Commun*, 447(2):292–298, May 2014.

23. H.D. Campbell, M. Kamei, C. Claudianos, E. Woollatt, G.R. Sutherland, Y. Suzuki, M. Hida, S. Sugano, and I.G. Young. Human and mouse homologues of the *Drosophila melanogaster* tweety (tty) gene: a novel gene family encoding predicted transmembrane proteins. *Genomics*, 68(1):89–92, Aug 2000.

24. Q. Cao, J. Chen, L. Zhu, Y. Liu, Z. Zhou, J. Sha, S. Wang, and J. Li. A testis-specific and testis developmentally regulated tumor protein D52 (TPD52)-like protein TPD52L3/hD55 interacts with TPD52 family proteins. *Biochem Biophys Res Commun*, 344(3):798–806, Jun 2006.

25. J.C. Cardoso, R.C. Felix, and D.M. Power. Nematode and arthropod genomes provide new insights into the evolution of class 2 B1 GPCRs. *PLoS One*, 9(3):e92220, 2014.

26. M.E. Casad, D. Abraham, I.M. Kim, S. Frangakis, B. Dong, N. Lin, M.J. Wolf, and H.A. Rockman. Cardiomyopathy is associated with ribosomal protein gene haplo-insufficiency in *Drosophila melanogaster*. *Genetics*, 189(3):861–870, Nov 2011.

27. R.M. Ceinos, E. Torres-Nunez, R. Chamorro, B. Novoa, A. Figueras, N.M. Ruane, and J. Rotllant. Critical role of the matricellular protein SPARC in mediating erythroid progenitor cell development in zebrafish. *Cells Tissues Organs*, 197(3):196–208, 2013.

28. S. Chang, N.V. Mandalaywala, R.G. Snyder, M.C. Levendusky, and R.E. Dearborn, Jr. Hedgehog-dependent down-regulation of the tumor suppressor, vitamin D3 up-regulated protein 1 (VDUP1), precedes lamina development in Drosophila. *Brain Res*, 1324:1–13, Apr 2010.

29. S. Charrin, S. Jouannet, C. Boucheix, and E. Rubinstein. Tetraspanins at a glance. *J Cell Sci*, 127(Pt 17):3641–3648, Sep 2014.

30. L. Chen, M. Krause, M. Sepanski, and A. Fire. The Caenorhabditis elegans MYOD homologue HLH-1 is essential for proper muscle function and complete morphogenesis. *Development*, 120(6):1631–1641, Jun 1994.

31. M. Chen, R.N. Martins, and M. Lardelli. Complex splicing and neural expression of duplicated tau genes in zebrafish embryos. *J Alzheimers Dis*, 18(2):305–317, 2009.

32. W.W. Chen, K. Birsoy, M.M. Mihaylova, H. Snitkin, I. Stasinski, B. Yucel, E.C. Bayraktar, J.E. Carette, C.B. Clish, T.R. Brummelkamp, D.D. Sabatini, and D.M. Sabatini. Inhibition of ATPIF1 ameliorates severe mitochondrial respiratory chain dysfunction in mammalian cells. *Cell Rep*, 7(1):27–34, Apr 2014.

33. H. Cheng, J.A. Govindan, and D. Greenstein. Regulated trafficking of the MSP/Eph receptor during oocyte meiotic maturation in *C. elegans*. *Curr Biol*, 18(10):705–714, May 2008.

34. M.C. Chicka and E.R. Chapman. Concurrent binding of complexin and synaptotagmin to liposome-embedded SNARE complexes. *Biochemistry*, 48(4):657–659, Feb 2009.

35. L.T. Chien and H.C. Hartzell. Drosophila bestrophin-1 chloride current is dually regulated by calcium and cell volume. *J Gen Physiol*, 130(5):513–524, Nov 2007.

36. L.T. Chien and H.C. Hartzell. Rescue of volume-regulated anion current by bestrophin mutants with altered charge selectivity. *J Gen Physiol*, 132(5):537–546, Nov 2008.

37. M. Chiodin, J.G. Achatz, A. Wanninger, and P. Martinez. Molecular architecture of muscles in an acoel and its evolutionary implications. *J Exp Zool B Mol Dev Evol*, 316(6):427–439, Sep 2011.

38. J.J. Chua, E. Butkevich, J.M. Worseck, M. Kittelmann, M. Gronborg, E. Behrmann, U. Stelzl, N.J. Pavlos, M.M. Lalowski, S. Eimer, E.E. Wanker, D.R. Klopfenstein, and R. Jahn. Phosphorylation-regulated axonal dependent transport of syntaxin 1 is mediated by a Kinesin-1 adapter. *Proc Natl Acad Sci U S A*, 109(15):5862–5867, Apr 2012.

39. P.C. Chung, W.S. Lin, P.J. Scotting, F.Y. Hsieh, H.L. Wu, and Y.C. Cheng. Zebrafish Her8a is activated by Su(H)-dependent Notch signaling and is essential for the inhibition of neurogenesis. *PLoS One*, 6(4):e19394, 2011.

40. A. Claperon and M. Therrien. KSR and CNK: two scaffolds regulating RAS-mediated RAF activation. *Oncogene*, 26(22):3143–3158, May 2007.

41. B.M. Cleveland and J.P. Evenhuis. Molecular characterization of atrogin-1/F-box protein-32 (FBXO32) and F-box protein-25 (FBXO25) in rainbow trout (*Oncorhynchus mykiss*): Expression across tissues in response to feed deprivation. *Comp Biochem Physiol B Biochem Mol Biol*, 157(3):248–257, Nov 2010.

42. T.A. Craig, S. Sommer, C.R. Sussman, J.P. Grande, and R. Kumar. Expression and regulation of the vitamin D receptor in the zebrafish, *Danio rerio*. *J Bone Miner Res*, 23(9):1486–1496, Sep 2008.

43. P. Dai, S.Y. Jeong, Y. Yu, T. Leng, W. Wu, L. Xie, and X. Chen. Modulation of TLR signaling by multiple MyD88-interacting partners including leucine-rich repeat Fli-I-interacting proteins. *J Immunol*, 182(6):3450–3460, Mar 2009.

44. A.S. Denes, G. Jekely, P.R. Steinmetz, F. Raible, H. Snyman, B. Prud'homme, D.E. Ferrier, G. Balavoine, and D. Arendt. Molecular architecture of annelid nerve cord supports common origin of nervous system centralization in bilateria. *Cell*, 129(2):277–288, Apr 2007.

45. T. Dickmeis, C. Plessy, S. Rastegar, P. Aanstad, R. Herwig, F. Chalmel, N. Fischer, and U. Strahle. Expression profiling and comparative genomics identify a conserved regulatory region controlling midline expression in the zebrafish embryo. *Genome Res*, 14(2):228–238, Feb 2004.

46. C.Q. Doe, Q. Chu-LaGraff, D.M. Wright, and M.P. Scott. The prospero gene specifies cell fates in the Drosophila central nervous system. *Cell*, 65(3):451–464, May 1991.

47. A.M. Dolga, A. de Andrade, L. Meissner, H.G. Knaus, M. Hollerhage, P. Christophersen, H. Zischka, N. Plesnila, G.U. Hoglinger, and C. Culmsee. Subcellular expression and neuroprotective effects of SK channels in human dopaminergic neurons. *Cell Death Dis*, 5:e999, 2014.

48. T. Domazet-Loso and D. Tautz. A phylogenetically based transcriptome age index mirrors ontogenetic divergence patterns. *Nature*, 468(7325):815–818, Dec 2010.

49. J. Droge and W. Makalowski. Phylogenetic analysis reveals wide distribution of globin X. *Biol Direct*, 6:54, 2011.

50. J.S. Duerr, D.L. Frisby, J. Gaskin, A. Duke, K. Asermely, D. Huddleston, L.E. Eiden, and J.B. Rand. The cat-1 gene of *Caenorhabditis elegans* encodes a vesicular monoamine transporter required for specific monoamine-dependent behaviors. *J Neurosci*, 19(1):72–84, Jan 1999.

51. J.S. Duerr, H.P. Han, S.D. Fields, and J.B. Rand. Identification of major classes of cholinergic neurons in the nematode *Caenorhabditis elegans*. *J Comp Neurol*, 506(3):398–408, Jan 2008.

52. L. Echevarria, P. Clemente, R. Hernandez-Sierra, M.E. Gallardo, M.A. Fernandez-Moreno, and R. Garesse. Glutamyl-tRNAGln amidotransferase is essential for mammalian mitochondrial translation in vivo. *Biochem J*, 460(1):91–101, May 2014.

53. B.M. Edens, S. Ajroud-Driss, L. Ma, and Y.C. Ma. Molecular mechanisms and animal models of spinal muscular atrophy. *Biochim Biophys Acta*, Aug 2014.

54. D.G. Edmondson and E.N. Olson. A gene with homology to the myc similarity region of MyoD1 is expressed during myogenesis and is sufficient to activate the muscle differentiation program. *Genes Dev*, 3(5):628–640, May 1989.

55. C. Fatouros, G.J. Pir, J. Biernat, S.P. Koushika, E. Mandelkow, E.M. Mandelkow, E. Schmidt, and R. Baumeister. Inhibition of tau aggregation in a novel *Caenorhabditis elegans* model of tauopathy mitigates proteotoxicity. *Hum Mol Genet*, 21(16):3587–3603, Aug 2012.

56. I. Fernandes, Y. Bastien, T. Wai, K. Nygard, R. Lin, O. Cormier, H.S. Lee, F. Eng, N.R. Bertos, N. Pelletier, S. Mader, V.K. Han, X.J. Yang, and J.H. White. Ligand-dependent nuclear receptor corepressor LCoR functions by histone deacetylase-dependent and -independent mechanisms. *Mol Cell*, 11(1):139–150, Jan 2003.

57. A.L. Fisher and M. Caudy. Groucho proteins: transcriptional corepressors for specific subsets of DNA-binding transcription factors in vertebrates and invertebrates. *Genes Dev*, 12(13):1931–1940, Jul 1998.

58. M.C. Fitzgerald and J.E. Schwarzbauer. Importance of the basement membrane protein SPARC for viability and fertility in *Caenorhabditis elegans*. *Curr Biol*, 8(23):1285–1288, Nov 1998.

59. M.L. Florez-McClure, L.A. Hohsfield, G. Fonte, M.T. Bealor, and C.D. Link. Decreased insulin-receptor signaling promotes the autophagic degradation of beta-amyloid peptide in *C. elegans*. *Autophagy*, 3(6):569–580, Nov 2007.

60. A.R. Frand, S. Russel, and G. Ruvkun. Functional genomic analysis of *C. elegans* molting. *PLoS Biol*, 3(10):e312, Oct 2005.

61. C.Y. Fu, H.C. Lee, and H.J. Tsai. The molecular structures and expression patterns of zebrafish troponin I genes. *Gene Expr Patterns*, 9(5):348–356, Jun 2009.

62. T.A. Fulga, I. Elson-Schwab, V. Khurana, M.L. Steinhilb, T.L. Spires, B.T. Hyman, and M.B. Feany. Abnormal bundling and accumulation of f-actin mediates tau-induced neuronal degeneration in vivo. *Nat Cell Biol*, 9(2):139–148, Feb 2007.

63. S.E. George, K. Simokat, J. Hardin, and A.D. Chisholm. The VAB-1 Eph receptor tyrosine kinase functions in neural and epithelial morphogenesis in *C. elegans*. *Cell*, 92(5):633–643, Mar 1998.

64. A.R. Gerhold, D.J. Richter, A.S. Yu, and I.K. Hariharan. Identification and characterization of genes required for compensatory growth in Drosophila. *Genetics*, 189(4):1309–1326, Dec 2011.

65. F.B. Gertler, K.K. Hill, M.J. Clark, and F.M. Hoffmann. Dosage-sensitive modifiers of Drosophila abl tyrosine kinase function: prospero, a regulator of axonal outgrowth, and disabled, a novel tyrosine kinase substrate. *Genes Dev*, 7(3):441–453, Mar 1993.

66. N. Giagtzoglou, P. Alifragis, K.A. Koumbanakis, and C. Delidakis. Two modes of recruitment of E(spl) repressors onto target genes. *Development*, 130(2):259–270, Jan 2003.

67. J.G. Gindhart, J. Chen, M. Faulkner, R. Gandhi, K. Doerner, T. Wisniewski, and A. Nandlestadt. The kinesin-associated protein UNC-76 is required for axonal transport in the Drosophila nervous system. *Mol Biol Cell*, 14(8):3356–3365, Aug 2003.

68. V. Ginet, M.P. Pittet, C. Rummel, M.C. Osterheld, R. Meuli, P.G. Clarke, J. Puyal, and A.C. Truttmann. Dying neurons in thalamus of asphyxiated term newborns and rats are autophagic. *Ann Neurol*, 76(5):695–711, Nov 2014.

69. S. Gluderer, E. Brunner, M. Germann, V. Jovaisaite, C. Li, C.A. Rentsch, E. Hafen, and H. Stocker. Madm (Mlf1 adapter molecule) cooperates with Bunched A to promote growth in Drosophila. *J Biol*, 9(1):9, 2010.

70. D. Glynn, R.J. Sizemore, and A.J. Morton. Early motor development is abnormal in complexin 1 knockout mice. *Neurobiol Dis*, 25(3):483–495, Mar 2007.

71. T.D. Goldman and M.N. Arbeitman. Genomic and functional studies of Drosophila sex hierarchy regulated gene expression in adult head and nervous system tissues. *PLoS Genet*, 3(11):e216, Nov 2007.

72. M.D. Gomes, S.H. Lecker, R.T. Jagoe, A. Navon, and A.L. Goldberg. Atrogin-1, a muscle-specific F-box protein highly expressed during muscle atrophy. *Proc Natl Acad Sci U S A*, 98(25):14440–14445, Dec 2001.

73. A.M. Gordon, E. Homsher, and M. Regnier. Regulation of contraction in striated muscle. *Physiol Rev*, 80(2):853–924, Apr 2000.

74. L. Goutebroze, E. Brault, C. Muchardt, J. Camonis, and G. Thomas. Cloning and characterization of SCHIP-1, a novel protein interacting specifically with spliced isoforms and naturally occurring mutant NF2 proteins. *Mol Cell Biol*, 20(5):1699–1712, Mar 2000.

75. B.R. Graveley, A.N. Brooks, J.W. Carlson, M.O. Duff, J.M. Landolin, L. Yang, C.G. Artieri, M.J. van Baren, N. Boley, B.W. Booth, J.B. Brown, L. Cherbas, C.A. Davis, A. Dobin, R. Li, W. Lin, J.H. Malone, N.R. Mattiuzzo, D. Miller, D. Sturgill, B.B. Tuch, C. Zaleski, D. Zhang, M. Blanchette, S. Dudoit, B. Eads, R.E. Green, A. Hammonds, L. Jiang, P. Kapranov, L. Langton, N. Perrimon, J.E. Sandler, K.H. Wan, A. Willingham, Y. Zhang, Y. Zou, J. Andrews, P.J. Bickel, S.E. Brenner, M.R. Brent, P. Cherbas, T.R. Gingeras, R.A. Hoskins, T.C. Kaufman, B. Oliver, and S.E. Celniker. The developmental transcriptome of *Drosophila melanogaster*. *Nature*, 471(7339):473–479, Mar 2011.

76. B. Guo, X. Huang, P. Zhang, L. Qi, Q. Liang, X. Zhang, J. Huang, B. Fang, W. Hou, J. Han, and H. Zhang. Genome-wide screen identifies signaling pathways that regulate autophagy during *Caenorhabditis elegans* development. *EMBO Rep*, 15(6):705–713, Jun 2014.

77. S. Han, S. Bahmanyar, P. Zhang, N. Grishin, K. Oegema, R. Crooke, M. Graham, K. Reue, J.E. Dixon, and J.M. Goodman. Nuclear envelope phosphatase 1-regulatory subunit 1 (formerly TMEM188) is the metazoan Spo7p ortholog and functions in the lipin activation pathway. *J Biol Chem*, 287(5):3123–3137, Jan 2012.

78. B.P. Head, M. Zulaika, S. Ryazantsev, and A.M. van der Bliek. A novel mitochondrial outer membrane protein, MOMA-1, that affects cristae morphology in *Caenorhabditis elegans*. *Mol Biol Cell*, 22(6):831–841, Mar 2011.

79. G. Heidary and M.E. Fortini. Identification and characterization of the Drosophila tau homolog. *Mech Dev*, 108(1-2):171–178, Oct 2001.

80. R. Herranz, J. Mateos, J.A. Mas, E. Garcia-Zaragoza, M. Cervera, and R. Marco. The coevolution of insect muscle TpnT and TpnI gene isoforms. *Mol Biol Evol*, 22(11):2231–2242, Nov 2005.

81. R.J. Hobson, Q. Liu, S. Watanabe, and E.M. Jorgensen. Complexin maintains vesicles in the primed state in *C. elegans*. *Curr Biol*, 21(2):106–113, Jan 2011.

82. S. Hokari, K. Miura, I. Koyama, M. Kobayashi, S. Komine, and T. Komoda. A restriction endonuclease assay for expression of human alpha-amylase isozymes. *Clin Chim Acta*, 322(1-2):113–116, Aug 2002.

83. L.Z. Holland, J.E. Carvalho, H. Escriva, V. Laudet, M. Schubert, S.M. Shimeld, and J.K. Yu. Evolution of bilaterian central nervous systems: a single origin? *Evodevo*, 4(1):27, 2013.

84. P.C. Hopkins. Neurodegeneration in a Drosophila model for the function of TMCC2, an amyloid protein precursor-interacting and apolipoprotein E-binding protein. *PLoS One*, 8(2):e55810, 2013.

85. B.E. Housden, A.Q. Fu, A. Krejci, F. Bernard, B. Fischer, S. Tavare, S. Russell, and S.J. Bray. Transcriptional dynamics elicited by a short pulse of notch activation involves feed-forward regulation by E(spl)/Hes genes. *PLoS Genet*, 9(1):e1003162, 2013.

86. S.M. Houten, S. Denis, H. Te Brinke, A. Jongejan, A.H. van Kampen, E.J. Bradley, F. Baas, R.C. Hennekam, D.S. Millington, S.P. Young, D.M. Frazier, M. Gucsavas-Calikoglu, and R.J. Wanders. Mitochondrial NADP(H) deficiency due to a mutation in NADK2 causes dienoyl-CoA reductase deficiency with hyperlysinemia. *Hum Mol Genet*, 23(18):5009–5016, Sep 2014.

87. D. Houzelstein, I.R. Goncalves, A.J. Fadden, S.S. Sidhu, D.N. Cooper, K. Drickamer, H. Leffler, and F. Poirier. Phylogenetic analysis of the vertebrate galectin family. *Mol Biol Evol*, 21(7):1177–1187, Jul 2004.

88. C.D. Hsiao, W.Y. Tsai, L.S. Horng, and H.J. Tsai. Molecular structure and developmental expression of three muscle-type troponin T genes in zebrafish. *Dev Dyn*, 227(2):266–279, Jun 2003.

89. S.Y. Hsu, Y.J. Chen, and P. Ouyang. Pnn and SR family proteins are differentially expressed in mouse central nervous system. *Histochem Cell Biol*, 135(4):361–373, Apr 2011.

90. S.Y. Hsu, Y.C. Cheng, H.Y. Shih, and P. Ouyang. Dissection of the role of Pinin in the development of zebrafish posterior pharyngeal cartilages. *Histochem Cell Biol*, 138(1):127–140, Jul 2012.

91. Z. Hu, D. Dandekar, P.J. O'Shaughnessy, K. De Gendt, G. Verhoeven, and M.F. Wilkinson. Androgen-induced Rhox homeobox genes modulate the expression of AR-regulated genes. *Mol Endocrinol*, 24(1):60–75, Jan 2010.

92. D. Huang, R. Meier, P.A. Todd, and L.M. Chou. Slow mitochondrial COI sequence evolution at the base of the metazoan tree and its implications for DNA barcoding. *J Mol Evol*, 66(2):167–174, Feb 2008.

93. S. Huang, S. Yuan, M. Dong, J. Su, C. Yu, Y. Shen, X. Xie, Y. Yu, X. Yu, S. Chen, S. Zhang, P. Pontarotti, and A. Xu. The phylogenetic analysis of tetraspanins projects the evolution of cell-cell interactions from unicellular to multicellular organisms. *Genomics*, 86(6):674–684, Dec 2005.

94. T. Hummel, K. Krukkert, J. Roos, G. Davis, and C. Klambt. Drosophila Futsch/22C10 is a MAP1B-like protein required for dendritic and axonal development. *Neuron*, 26(2):357–370, May 2000.

95. S. Huntwork and J.T. Littleton. A complexin fusion clamp regulates spontaneous neurotransmitter release and synaptic growth. *Nat Neurosci*, 10(10):1235–1237, Oct 2007.

96. N. Ichikawa, C. Ando, and M. Fumino. *Caenorhabditis elegans* MAI-1 protein, which is similar to mitochondrial ATPase inhibitor (IF1), can inhibit yeast F0F1-ATPase but cannot be transported to yeast mitochondria. *J Bioenerg Biomembr*, 38(2):93–99, Apr 2006.

97. S. Ihara, E.J. Hagedorn, M.A. Morrissey, Q. Chi, F. Motegi, J.M. Kramer, and D.R. Sherwood. Basement membrane sliding and targeted adhesion remodels tissue boundaries during uterine-vulval attachment in *Caenorhabditis elegans*. *Nat Cell Biol*, 13(6):641–651, Jun 2011.

98. K. Iijima-Ando, M. Sekiya, A. Maruko-Otake, Y. Ohtake, E. Suzuki, B. Lu, and K.M. Iijima. Loss of axonal mitochondria promotes tau-mediated neurodegeneration and Alzheimer's disease-related tau phosphorylation via PAR-1. *PLoS Genet*, 8(8):e1002918, 2012.

99. J.W. Jang, W.Y. Lee, J.H. Lee, S.H. Moon, C.H. Kim, and H.M. Chung. A novel Fbxo25 acts as an E3 ligase for destructing cardiac specific transcription factors. *Biochem Biophys Res Commun*, 410(2):183–188, Jul 2011.

100. G. Jekely. Global view of the evolution and diversity of metazoan neuropeptide signaling. *Proc Natl Acad Sci U S A*, 110(21):8702–8707, May 2013.

101. L. Jiang and S.T. Crews. Dysfusion transcriptional control of Drosophila tracheal migration, adhesion, and fusion. *Mol Cell Biol*, 26(17):6547–6556, Sep 2006.

102. L. Jiang, J.C. Pearson, and S.T. Crews. Diverse modes of Drosophila tracheal fusion cell transcriptional regulation. *Mech Dev*, 127(5-6):265–280, May 2010.

103. S. Jin, L. Pan, Z. Liu, Q. Wang, Z. Xu, and Y.Q. Zhang. Drosophila Tubulin-specific chaperone E functions at neuromuscular synapses and is required for microtubule network formation. *Development*, 136(9):1571–1581, May 2009.

104. J.H. Joo, T.J. Taxter, G.C. Munguba, Y.H. Kim, K. Dhaduvai, N.W. Dunn, W.J. Degan, S.P. Oh, and S.P. Sugrue. Pinin modulates expression of an intestinal homeobox gene, Cdx2, and plays an essential role for small intestinal morphogenesis. *Dev Biol*, 345(2):191–203, Sep 2010.

105. T. Kanome, N. Itoh, F. Ishikawa, K. Mori, J.R. Kim-Kaneyama, K. Nose, and M. Shibanuma. Characterization of Jumping translocation breakpoint (JTB) gene product isolated as a TGF-beta1-inducible clone involved in regulation of mitochondrial function, cell growth and cell death. *Oncogene*, 26(41):5991–6001, Sep 2007.

106. E.D. Karousis and D.C. Sideris. A subtle alternative splicing event gives rise to a widely expressed human RNase k isoform. *PLoS One*, 9(5):e96557, 2014.

107. J. Kim, S. Lee, M. Hwang, S. Ko, C. Min, and J. Kim-Ha. Bunched specifically regulates alpha/beta mushroom body neuronal cell proliferation during metamorphosis. *Neuroscience*, 161(1):46–52, Jun 2009.

108. J.D. Kim, H. Kang, B. Larrivee, M.Y. Lee, M. Mettlen, S.L. Schmid, B.L. Roman, Y. Qyang, A. Eichmann, and S.W. Jin. Context-dependent proangiogenic function of bone morphogenetic protein signaling is mediated by disabled homolog 2. *Dev Cell*, 23(2):441–448, Aug 2012.

109. Y.I. Kim, S. Lee, S.H. Jung, H.T. Kim, J.H. Choi, M.S. Lee, K.H. You, S.Y. Yeo, K.W. Yoo, S. Kwak, J.N. Lee, R. Park, S.K. Choe, and C.H. Kim. Establishment of a bone-specific col10a1:GFP transgenic zebrafish. *Mol Cells*, 36(2):145–150, Aug 2013.

110. J. Kitano, Y. Yamazaki, K. Kimura, T. Masukado, Y. Nakajima, and S. Nakanishi. Tamalin is a scaffold protein that interacts with multiple neuronal proteins in distinct modes of protein-protein association. *J Biol Chem*, 278(17):14762–14768, Apr 2003.

111. T. Komori. Regulation of bone development and extracellular matrix protein genes by RUNX2. *Cell Tissue Res*, 339(1):189–195, Jan 2010.

112. M. Kostrouchova, M. Krause, Z. Kostrouch, and J.E. Rall. Nuclear hormone receptor CHR3 is a critical regulator of all four larval molts of the nematode *Caenorhabditis elegans*. *Proc Natl Acad Sci U S A*, 98(13):7360–7365, Jun 2001.

113. N.A. Kouns, J. Nakielna, F. Behensky, M.W. Krause, Z. Kostrouch, and M. Kostrouchova. NHR-23 dependent collagen and hedgehog-related genes required for molting. *Biochem Biophys Res Commun*, 413(4):515–520, Oct 2011.

114. B.C. Kraemer and G.D. Schellenberg. SUT-1 enables tau-induced neurotoxicity in *C. elegans*. *Hum Mol Genet*, 16(16):1959–1971, Aug 2007.

115. A.J. Krol, D. Roellig, M.L. Dequeant, O. Tassy, E. Glynn, G. Hattem, A. Mushegian, A.C. Oates, and O. Pourquie. Evolutionary plasticity of segmentation clock networks. *Development*, 138(13):2783–2792, Jul 2011.

116. R. Labrecque, C. Vigneault, P. Blondin, and M.A. Sirard. Gene expression analysis of bovine oocytes at optimal coasting time combined with GnRH antagonist during the no-FSH period. *Theriogenology*, 81(8):1092–1100, May 2014.

117. M.C. Lagerstrom and H.B. Schioth. Structural diversity of G protein-coupled receptors and significance for drug discovery. *Nat Rev Drug Discov*, 7(4):339–357, Apr 2008.

118. M. Lagha, J.P. Bothma, E. Esposito, S. Ng, L. Stefanik, C. Tsui, J. Johnston, K. Chen, D.S. Gilmour, J. Zeitlinger, and M.S. Levine. Paused Pol II coordinates tissue morphogenesis in the Drosophila embryo. *Cell*, 153(5):976–987, May 2013.

119. D.C. Lanza, D.M. Trindade, E.M. Assmann, and J. Kobarg. Over-expression of GFP-FEZ1 causes generation of multi-lobulated nuclei mediated by microtubules in HEK293 cells. *Exp Cell Res*, 314(10):2028–2039, Jun 2008.

120. M. Larhammar, K. Patra, M. Blunder, L. Emilsson, C. Peuckert, E. Arvidsson, D. Ronnlund, J. Preobraschenski, C. Birgner, C. Limbach, J. Widengren, H. Blom, R. Jahn, A. Wallen-Mackenzie, and K. Kullander. SLC10A4 is a vesicular amine-associated transporter modulating dopamine homeostasis. *Biol Psychiatry*, 77(6):526–536, Mar 2015.

121. K.S. Lee and B. Lu. The myriad roles of Miro in the nervous system: axonal transport of mitochondria and beyond. *Front Cell Neurosci*, 8:330, 2014.

122. R. Levayer and E. Moreno. Mechanisms of cell competition: themes and variations. *J Cell Biol*, 200(6):689–698, Mar 2013.

123. M.C. Levendusky, J. Basle, S. Chang, N.V. Mandalaywala, J.M. Voigt, and R.E. Dearborn, Jr. Expression and regulation of vitamin D3 upregulated protein 1 (VDUP1) is conserved in mammalian and insect brain. *J Comp Neurol*, 517(5):581–600, Dec 2009.

124. M. Levin, T. Hashimshony, F. Wagner, and I. Yanai. Developmental milestones punctuate gene expression in the Caenorhabditis embryo. *Dev Cell*, 22(5):1101–1108, May 2012.

125. C.Y. Lin, H.C. Lee, H.C. Chen, C.C. Hsieh, and H.J. Tsai. Normal function of Myf5 during gastrulation is required for pharyngeal arch cartilage development in zebrafish embryos. *Zebrafish*, 10(4):486–499, Dec 2013.

126. J.X. Liu, B. Hu, Y. Wang, J.F. Gui, and W. Xiao. Zebrafish eaf1 and eaf2/u19 mediate effective convergence and extension movements through the maintenance of wnt11 and wnt5 expression. *J Biol Chem*, 284(24):16679–16692, Jun 2009.

127. J.X. Liu, D. Zhang, X. Xie, G. Ouyang, X. Liu, Y. Sun, and W. Xiao. Eaf1 and Eaf2 negatively regulate canonical Wnt/beta-catenin signaling. *Development*, 140(5):1067–1078, Mar 2013.

128. L. Liu, D. Feng, G. Chen, M. Chen, Q. Zheng, P. Song, Q. Ma, C. Zhu, R. Wang, W. Qi, L. Huang, P. Xue, B. Li, X. Wang, H. Jin, J. Wang, F. Yang, P. Liu, Y. Zhu, S. Sui, and Q. Chen. Mitochondrial outer-membrane protein FUNDC1 mediates hypoxia-induced mitophagy in mammalian cells. *Nat Cell Biol*, 14(2):177–185, Feb 2012.

129. F.N. Lolo, S. Casas-Tinto, and E. Moreno. Cell competition time line: winners kill losers, which are extruded and engulfed by hemocytes. *Cell Rep*, 2(3):526–539, Sep 2012.

130. Y. Lv, K. Zhang, and H. Gao. Paip1, an effective stimulator of translation initiation, is targeted by WWP2 for ubiquitination and degradation. *Mol Cell Biol*, 34(24):4513–4522, Dec 2014.

131. X. Ma and J.X. Liu. Eafs control erythroid cell fate by regulating c-myb expression through Wnt signaling. *PLoS One*, 8(5):e64576, 2013.

132. M.C. Marin, J.R. Rodriguez, and A. Ferrus. Transcription of Drosophila troponin i gene is regulated by two conserved, functionally identical, synergistic elements. *Mol Biol Cell*, 15(3):1185–1196, Mar 2004.

133. J.A. Martin, Z. Hu, K.M. Fenz, J. Fernandez, and J.S. Dittman. Complexin has opposite effects on two modes of synaptic vesicle fusion. *Curr Biol*, 21(2):97–105, Jan 2011.

134. P.M. Martin, M. Carnaud, G. Garcia del Cano, M. Irondelle, T. Irinopoulou, J.A. Girault, B. Dargent, and L. Goutebroze. Schwannomin-interacting protein-1 isoform IQCJ-SCHIP-1 is a late component of nodes of Ranvier and axon initial segments. *J Neurosci*, 28(24):6111–6117, Jun 2008.

135. N. Martinek, J. Shahab, M. Saathoff, and M. Ringuette. Haemocyte-derived SPARC is required for collagen-IV-dependent stability of basal laminae in Drosophila embryos. *J Cell Sci*, 121(Pt 10):1671–1680, May 2008.

136. S.J. Marygold, J. Roote, G. Reuter, A. Lambertsson, M. Ashburner, G.H. Millburn, P.M. Harrison, Z. Yu, N. Kenmochi, T.C. Kaufman, S.J. Leevers, and K.R. Cook. The ribosomal protein genes and Minute loci of *Drosophila melanogaster*. *Genome Biol*, 8(10):R216, 2007.

137. J.A. Mas, E. Garcia-Zaragoza, and M. Cervera. Two functionally identical modular enhancers in Drosophila troponin T gene establish the correct protein levels in different muscle types. *Mol Biol Cell*, 15(4):1931–1945, Apr 2004.

138. C.A. Matthews, J.E. Shaw, J.A. Hooper, I.G. Young, M.F. Crouch, and H.D. Campbell. Expression and evolution of the mammalian brain gene Ttyh1. *J Neurochem*, 100(3):693–707, Feb 2007.

139. A.D. Maturana, T. Fujita, and S. Kuroda. Functions of fasciculation and elongation protein zeta-1 (FEZ1) in the brain. *ScientificWorldJournal*, 10:1646–1654, 2010.

140. M. Mazzocco, M. Maffei, A. Egeo, A. Vergano, P. Arrigo, R. Di Lisi, F. Ghiotto, and P. Scartezzini. The identification of a novel human homologue of the SH3 binding glutamic acid-rich (SH3BGR) gene establishes a new family of highly conserved small proteins related to Thioredoxin Superfamily. *Gene*, 291(1-2):233–239, May 2002.

141. S. McNabb, S. Greig, and T. Davis. The alcohol dehydrogenase gene is nested in the outspread locus of *Drosophila melanogaster*. *Genetics*, 143(2):897–911, Jun 1996.

142. T.J. Melia, Jr. Putting the clamps on membrane fusion: how complexin sets the stage for calcium-mediated exocytosis. *FEBS Lett*, 581(11):2131–2139, May 2007.

143. A.M. Michelson, S.M. Abmayr, M. Bate, A.M. Arias, and T. Maniatis. Expression of a MyoD family member prefigures muscle pattern in Drosophila embryos. *Genes Dev*, 4(12A):2086–2097, Dec 1990.

144. S. Miehe, A. Bieberstein, I. Arnould, O. Ihdene, H. Rutten, and C. Strubing. The phospholipid-binding protein SESTD1 is a novel regulator of the transient receptor potential channels TRPC4 and TRPC5. *J Biol Chem*, 285(16):12426–12434, Apr 2010.

145. O. Mirabeau and J.S. Joly. Molecular evolution of peptidergic signaling systems in bilaterians. *Proc Natl Acad Sci U S A*, 110(22):E2028–37, May 2013.

146. M. Misiak, S. Singh, S. Drewlo, C. Beyer, and S. Arnold. Brain region-specific vulnerability of astrocytes in response to 3-nitropropionic acid is mediated by cytochrome c oxidase isoform expression. *Cell Tissue Res*, 341(1):83–93, Jul 2010.

147. R.W. Mitchell and G.M. Hatch. Fatty acid transport into the brain: of fatty acid fables and lipid tails. *Prostaglandins Leukot Essent Fatty Acids*, 85(5):293–302, Nov 2011.

148. H. Moribe and E. Mekada. Co-occurrence of tetraspanin and ROS generators: Conservation in protein cross-linking and other developmental processes. *Worm*, 2(2):e23415, Apr 2013.

149. H. Moribe, J. Yochem, H. Yamada, Y. Tabuse, T. Fujimoto, and E. Mekada. Tetraspanin protein (TSP-15) is required for epidermal integrity in *Caenorhabditis elegans*. *J Cell Sci*, 117(Pt 22):5209–5220, Oct 2004.

150. X. Mou, D.M. Duncan, E.H. Baehrecke, and I. Duncan. Control of target gene specificity during metamorphosis by the steroid response gene E93. *Proc Natl Acad Sci U S A*, 109(8):2949–2954, Feb 2012.

151. V.S. Moulle, C. Cansell, S. Luquet, and C. Cruciani-Guglielmacci. The multiple roles of fatty acid handling proteins in brain. *Front Physiol*, 3:385, 2012.

152. C.D. Myers, P.Y. Goh, T.S. Allen, E.A. Bucher, and T. Bogaert. Developmental genetic analysis of troponin T mutations in striated and nonstriated muscle cells of *Caenorhabditis elegans*. *J Cell Biol*, 132(6):1061–1077, Mar 1996.

153. A. Nagao, T. Suzuki, T. Katoh, Y. Sakaguchi, and T. Suzuki. Biogenesis of glutaminyl-mt tRNAGln in human mitochondria. *Proc Natl Acad Sci U S A*, 106(38):16209–16214, Sep 2009.

154. B. Naimi, A. Harrison, M. Cummins, U. Nongthomba, S. Clark, I. Canal, A. Ferrus, and J.C. Sparrow. A tropomyosin-2 mutation suppresses a troponin I myopathy in Drosophila. *Mol Biol Cell*, 12(5):1529–1539, May 2001.

155. M. Nakamura, D. Baldwin, S. Hannaford, J. Palka, and C. Montell. Defective proboscis extension response (DPR), a member of the Ig superfamily required for the gustatory response to salt. *J Neurosci*, 22(9):3463–3472, May 2002.

156. V.H. Nguyen, B. Schmid, J. Trout, S.A. Connors, M. Ekker, and M.C. Mullins. Ventral and lateral regions of the zebrafish gastrula, including the neural crest progenitors, are established by a bmp2b/swirl pathway of genes. *Dev Biol*, 199(1):93–110, Jul 1998.

157. U. Nongthomba, M. Ansari, D. Thimmaiya, M. Stark, and J. Sparrow. Aberrant splicing of an alternative exon in the Drosophila troponin-T gene affects flight muscle development. *Genetics*, 177(1):295–306, Sep 2007.

158. T.W. O'Brien. Evolution of a protein-rich mitochondrial ribosome: implications for human genetic disease. *Gene*, 286(1):73–79, Mar 2002.

159. K. Ohara, A. Enomoto, T. Kato, T. Hashimoto, M. Isotani-Sakakibara, N. Asai, M. Ishida-Takagishi, L. Weng, M. Nakayama, T. Watanabe, K. Kato, K. Kaibuchi, Y. Murakumo, Y. Hirooka, H. Goto, and M. Takahashi. Involvement of Girdin in the determination of cell polarity during cell migration. *PLoS One*, 7(5):e36681, 2012.

160. M. Okumura, A.M. Katsuyama, H. Shibata, and M. Maki. VPS37 isoforms differentially modulate the ternary complex formation of ALIX, ALG-2, and ESCRT-i. *Biosci Biotechnol Biochem*, 77(8):1715–1721, 2013.

161. G. Oliver, B. Sosa-Pineda, S. Geisendorf, E.P. Spana, C.Q. Doe, and P. Gruss. Prox 1, a prospero-related homeobox gene expressed during mouse development. *Mech Dev*, 44(1):3–16, Nov 1993.

162. H. Ota, T. Hikita, T. Nishioka, M. Matsumoto, J. Ito, N. Asai, A. Enomoto, M. Takahashi, K. Kaibuchi, K. Sobue, and K. Sawamoto. Proteomic analysis of Girdin-interacting proteins in migrating new neurons in the postnatal mouse brain. *Biochem Biophys Res Commun*, 442(1-2):16–21, Dec 2013.

163. H. Parikh, E. Nilsson, C. Ling, P. Poulsen, P. Almgren, H. Nittby, K.F. Eriksson, A. Vaag, and L.C. Groop. Molecular correlates for maximal oxygen uptake and type 1 fibers. *Am J Physiol Endocrinol Metab*, 294(6):E1152–9, Jun 2008.

164. S.E. Patterson, N.C. Bird, and S.H. Devoto. BMP regulation of myogenesis in zebrafish. *Dev Dyn*, 239(3):806–817, Mar 2010.

165. M. Pejznochova, M. Tesarova, H. Hansikova, M. Magner, T. Honzik, K. Vinsova, Z. Hajkova, V. Havlickova, and J. Zeman. Mitochondrial DNA content and expression of genes involved in mtDNA transcription, regulation and maintenance during human fetal development. *Mitochondrion*, 10(4):321–329, Jun 2010.

166. C. Picco, J. Scholz-Starke, A. Naso, V. Preger, F. Sparla, P. Trost, and A. Carpaneto. How are cytochrome b561 electron currents controlled by membrane voltage and substrate availability? *Antioxid Redox Signal*, 21(3):384–391, Jul 2014.

167. S.N. Popova and I. Alafuzoff. Distribution of SLC10A4, a synaptic vesicle protein in the human brain, and the association of this protein with Alzheimer's disease-related neuronal degeneration. *J Alzheimers Dis*, 37(3):603–610, 2013.

168. M. Portela, S. Casas-Tinto, C. Rhiner, J.M. Lopez-Gay, O. Dominguez, D. Soldini, and E. Moreno. Drosophila SPARC is a self-protective signal expressed by loser cells during cell competition. *Dev Cell*, 19(4):562–573, Oct 2010.

169. J.A. Powell-Coffman, J. Knight, and W.B. Wood. Onset of *C. elegans* gastrulation is blocked by inhibition of embryonic transcription with an RNA polymerase antisense RNA. *Dev Biol*, 178(2):472–483, Sep 1996.

170. G. Ranganayakulu, R.A. Schulz, and E.N. Olson. Wingless signaling induces nautilus expression in the ventral mesoderm of the Drosophila embryo. *Dev Biol*, 176(1):143–148, May 1996.

171. E. Reinstein, K. Orvin, E. Tayeb-Fligelman, H. Stiebel-Kalish, S. Tzur, A.L. Pimienta, L. Bazak, T. Bengal, L. Cohen, D.D. Gaton, C. Bormans, M. Landau, R. Kornowski, M. Shohat, and D.M. Behar. Mutations in TAX1BP3 cause dilated cardiomyopathy with septo-optic dysplasia. *Hum Mutat*, 36(4):439–442, Apr 2015.

172. M. Renaud, V. Praz, E. Vieu, L. Florens, M.P. Washburn, P. l'Hote, and N. Hernandez. Gene duplication and neofunctionalization: POLR3G and POLR3GL. *Genome Res*, 24(1):37–51, Jan 2014.

173. C. Rhiner, J.M. Lopez-Gay, D. Soldini, S. Casas-Tinto, F.A. Martin, L. Lombardia, and E. Moreno. Flower forms an extracellular code that reveals the fitness of a cell to its neighbors in Drosophila. *Dev Cell*, 18(6):985–998, Jun 2010.

174. C.E. Rocheleau, A. Ronnlund, S. Tuck, and M.V. Sundaram. *Caenorhabditis elegans* CNK-1 promotes Raf activation but is not essential for Ras/Raf signaling. *Proc Natl Acad Sci U S A*, 102(33):11757–11762, Aug 2005.

175. J. Roos, T. Hummel, N. Ng, C. Klambt, and G.W. Davis. Drosophila Futsch regulates synaptic microtubule organization and is necessary for synaptic growth. *Neuron*, 26(2):371–382, May 2000.

176. G.E. Roth, M.S. Gierl, L. Vollborn, M. Meise, R. Lintermann, and G. Korge. The Drosophila gene Start1: a putative cholesterol transporter and key regulator of ecdysteroid synthesis. *Proc Natl Acad Sci U S A*, 101(6):1601–1606, Feb 2004.

177. A.F. Ruaud, G. Lam, and C.S. Thummel. The Drosophila nuclear receptors DHR3 and betaFTZ-F1 control overlapping developmental responses in late embryos. *Development*, 137(1):123–131, Jan 2010.

178. E.I. Rugarli, E. Di Schiavi, M.A. Hilliard, S. Arbucci, C. Ghezzi, A. Facciolli, G. Coppola, A. Ballabio, and P. Bazzicalupo. The Kallmann syndrome gene homolog in *C. elegans* is involved in epidermal morphogenesis and neurite branching. *Development*, 129(5):1283–1294, Mar 2002.

179. B.M. Scheel and B. Hausdorf. Dynamic evolution of mitochondrial ribosomal proteins in Holozoa. *Mol Phylogenet Evol*, 76:67–74, Jul 2014.

180. J.E. Schwarzbauer and C.S. Spencer. The *Caenorhabditis elegans* homologue of the extracellular calcium binding protein SPARC/osteonectin affects nematode body morphology and mobility. *Mol Biol Cell*, 4(9):941–952, Sep 1993.

181. J. Schwendinger-Schreck, Y. Kang, and S.A. Holley. Modeling the zebrafish segmentation clock's gene regulatory network constrained by expression data suggests evolutionary transitions between oscillating and nonoscillating transcription. *Genetics*, 197(2):725–738, Jun 2014.

182. D.I. Shah, N. Takahashi-Makise, J.D. Cooney, L. Li, I.J. Schultz, E.L. Pierce, A. Narla, A. Seguin, S.M. Hattangadi, A.E. Medlock, N.B. Langer, T.A. Dailey, S.N. Hurst, D. Faccenda, J.M. Wiwczar, S.K. Heggers, G. Vogin, W. Chen, C. Chen, D.R. Campagna, C. Brugnara, Y. Zhou, B.L. Ebert, N.N. Danial, M.D. Fleming, D.M. Ward, M. Campanella, H.A. Dailey, J. Kaplan, and B.H. Paw. Mitochondrial Atpif1 regulates haem synthesis in developing erythroblasts. *Nature*, 491(7425):608–612, Nov 2012.

183. T.L. Shearer, M.J. Van Oppen, S.L. Romano, and G. Worheide. Slow mitochondrial DNA sequence evolution in the Anthozoa (Cnidaria). *Mol Ecol*, 11(12):2475–2487, Dec 2002.

184. T. Siegmund and M. Lehmann. The Drosophila Pipsqueak protein defines a new family of helix-turn-helix DNA-binding proteins. *Dev Genes Evol*, 212(3):152–157, Apr 2002.

185. J.S. Simske. Claudins reign: The claudin/EMP/PMP22/gamma channel protein family in *C. elegans*. *Tissue Barriers*, 1(3):e25502, Jul 2013.

186. J.S. Simske, M. Koppen, P. Sims, J. Hodgkin, A. Yonkof, and J. Hardin. The cell junction protein VAB-9 regulates adhesion and epidermal morphology in *C. elegans*. *Nat Cell Biol*, 5(7):619–625, Jul 2003.

187. E.R. Smith, B. Winter, J.C. Eissenberg, and A. Shilatifard. Regulation of the transcriptional activity of poised RNA polymerase II by the elongation factor ELL. *Proc Natl Acad Sci U S A*, 105(25):8575–8579, Jun 2008.

188. P. Smits, J.A. Smeitink, L.P. van den Heuvel, M.A. Huynen, and T.J. Ettema. Reconstructing the evolution of the mitochondrial ribosomal proteome. *Nucleic Acids Res*, 35(14):4686–4703, 2007.

189. J.K. Song, R. Kannan, G. Merdes, J. Singh, M. Mlodzik, and E. Giniger. Disabled is a bona fide component of the Abl signaling network. *Development*, 137(21):3719–3727, Nov 2010.

190. P.R. Steinmetz, J.E. Kraus, C. Larroux, J.U. Hammel, A. Amon-Hassenzahl, E. Houliston, G. Worheide, M. Nickel, B.M. Degnan, and U. Technau. Independent evolution of striated muscles in cnidarians and bilaterians. *Nature*, 487(7406):231–234, Jul 2012.

191. A.D. Stump, M. Dillon-White, and S. Gu. Molecular evolution of the moonlighting protein SMN in metazoans. *Comp Biochem Physiol Part D Genomics Proteomics*, 8(3):220–230, Sep 2013.

192. V. Subramaniam, P. Golik, D.G. Murdock, S. Levy, K.W. Kerstann, P.E. Coskun, G.A. Melkonian, and D.C. Wallace. MITOCHIP assessment of differential gene expression in the skeletal muscle of Ant1 knockout mice: coordinate regulation of OXPHOS, antioxidant, and apoptotic genes. *Biochim Biophys Acta*, 1777(7-8):666–675, Jul 2008.

193. H. Sun, T. Tsunenari, K.W. Yau, and J. Nathans. The vitelliform macular dystrophy protein defines a new family of chloride channels. *Proc Natl Acad Sci U S A*, 99(6):4008–4013, Mar 2002.

194. Y. Takashima, S. Kitaoka, T. Bando, and H. Kagawa. Expression profiles and unc-27 mutation rescue of the striated muscle type troponin I isoform-3 in *Caenorhabditis elegans*. *Genes Genet Syst*, 87(4):243–251, 2012.

195. S.J. Tapscott. The circuitry of a master switch: Myod and the regulation of skeletal muscle gene transcription. *Development*, 132(12):2685–2695, Jun 2005.

196. L. Teixeira. Whole-genome expression profile analysis of *Drosophila melanogaster* immune responses. *Brief Funct Genomics*, 11(5):375–386, Sep 2012.

197. K. Tessmar-Raible, F. Raible, F. Christodoulou, K. Guy, M. Rembold, H. Hausen, and D. Arendt. Conserved sensory-neurosecretory cell types in annelid and fish forebrain: insights into hypothalamus evolution. *Cell*, 129(7):1389–1400, Jun 2007.

198. M. Therrien, A.M. Wong, and G.M. Rubin. CNK, a RAF-binding multidomain protein required for RAS signaling. *Cell*, 95(3):343–353, Oct 1998.

199. H. Toda, H. Mochizuki, R. Flores, 3rd, R. Josowitz, T.B. Krasieva, V.J. Lamorte, E. Suzuki, J.G. Gindhart, K. Furukubo-Tokunaga, and T. Tomoda. UNC-51/ATG1 kinase regulates axonal transport by mediating motor-cargo assembly. *Genes Dev*, 22(23):3292–3307, Dec 2008.

200. E. Torres Nunez, C. Sobrino, P.J. Neale, R.M. Ceinos, S. Du, and J. Rotllant. Molecular response to ultraviolet radiation exposure in fish embryos: implications for survival and morphological development. *Photochem Photobiol*, 88(3):701–707, May 2012.

201. M. van Spronsen, M. Mikhaylova, J. Lipka, M.A. Schlager, D.J. van den Heuvel, M. Kuijpers, P.S. Wulf, N. Keijzer, J. Demmers, L.C. Kapitein, D. Jaarsma, H.C. Gerritsen, A. Akhmanova, and C.C. Hoogenraad. TRAK/Milton motor-adaptor proteins steer mitochondrial trafficking to axons and dendrites. *Neuron*, 77(3):485–502, Feb 2013.

202. D. Van Vactor, D.P. Wall, and K.G. Johnson. Heparan sulfate proteoglycans and the emergence of neuronal connectivity. *Curr Opin Neurobiol*, 16(1):40–51, Feb 2006.

203. L. Wang, Y. Sun, M. Jiang, and X. Zheng. Integrative decomposition procedure and Kappa statistics for the distinguished single molecular network construction and analysis. *J Biomed Biotechnol*, 2009:726728, 2009.

204. P. Wang, P.J. Lou, S. Leu, and P. Ouyang. Modulation of alternative pre-mRNA splicing in vivo by pinin. *Biochem Biophys Res Commun*, 294(2):448–455, Jun 2002.

205. Y. Wang, N. Kaneko, N. Asai, A. Enomoto, M. Isotani-Sakakibara, T. Kato, M. Asai, Y. Murakumo, H. Ota, T. Hikita, T. Namba, K. Kuroda, K. Kaibuchi, G.L. Ming, H. Song, K. Sawamoto, and M. Takahashi. Girdin is an intrinsic regulator of neuroblast chain migration in the rostral migratory stream of the postnatal brain. *J Neurosci*, 31(22):8109–8122, Jun 2011.

206. Z. Wang and R.G. Roeder. Three human RNA polymerase III-specific subunits form a subcomplex with a selective function in specific transcription initiation. *Genes Dev*, 11(10):1315–1326, May 1997.

207. K.J. Webb, M. Coolen, C.J. Gloeckner, C. Stigloher, B. Bahn, S. Topp, M. Ueffing, and L. Bally-Cuif. The enhancer of split transcription factor Her8a is a novel dimerisation partner for Her3 that controls anterior hindbrain neurogenesis in zebrafish. *BMC Dev Biol*, 11:27, 2011.

208. Q. Wei, Y. Rong, and B.M. Paterson. Stereotypic founder cell patterning and embryonic muscle formation in Drosophila require nautilus (MyoD) gene function. *Proc Natl Acad Sci U S A*, 104(13):5461–5466, Mar 2007.

209. R.C. Wong, S. Pollan, H. Fong, A. Ibrahim, E.L. Smith, M. Ho, A.L. Laslett, and P.J. Donovan. A novel role for an RNA polymerase III subunit POLR3G in regulating pluripotency in human embryonic stem cells. *Stem Cells*, 29(10):1517–1527, Oct 2011.

210. H.P. Wu, S.Y. Hsu, W.A. Wu, J.W. Hu, and P. Ouyang. Transgenic mice expressing mutant Pinin exhibit muscular dystrophy, nebulin deficiency and elevated expression of slow-type muscle fiber genes. *Biochem Biophys Res Commun*, 443(1):313–320, Jan 2014.

211. W. Wu, W. Tian, Z. Hu, G. Chen, L. Huang, W. Li, X. Zhang, P. Xue, C. Zhou, L. Liu, Y. Zhu, X. Zhang, L. Li, L. Zhang, S. Sui, B. Zhao, and D. Feng. ULK1 translocates to mitochondria and phosphorylates FUNDC1 to regulate mitophagy. *EMBO Rep*, 15(5):566–575, May 2014.

212. D. Yamazaki, Y. Tabara, S. Kita, H. Hanada, S. Komazaki, D. Naitou, A. Mishima, M. Nishi, H. Yamamura, S. Yamamoto, S. Kakizawa, H. Miyachi, S. Yamamoto, T. Miyata, Y. Kawano, K. Kamide, T. Ogihara, A. Hata, S. Umemura, M. Soma, N. Takahashi, Y. Imaizumi, T. Miki, T. Iwamoto, and H. Takeshima. TRIC-A channels in vascular smooth muscle contribute to blood pressure maintenance. *Cell Metab*, 14(2):231–241, Aug 2011.

213. T. Yamazaki, N. Fujiwara, H. Yukinaga, M. Ebisuya, T. Shiki, T. Kurihara, N. Kioka, T. Kambe, M. Nagao, E. Nishida, and S. Masuda. The closely related RNA helicases, UAP56 and URH49, preferentially form distinct mRNA export machineries and coordinately regulate mitotic progression. *Mol Biol Cell*, 21(16):2953–2965, Aug 2010.

214. X. Yang and B.N. Cheyette. SEC14 and spectrin domains 1 (Sestd1) and Dapper antagonist of catenin 1 (Dact1) scaffold proteins cooperatively regulate the Van Gogh-like 2 (Vangl2) four-pass transmembrane protein and planar cell polarity (PCP) pathway during embryonic development in mice. *J Biol Chem*, 288(28):20111–20120, Jul 2013.

215. U. Yavuzer, G.C. Smith, T. Bliss, D. Werner, and S.P. Jackson. DNA end-independent activation of DNA-PK mediated via association with the DNA-binding protein C1D. *Genes Dev*, 12(14):2188–2199, Jul 1998.

216. J.H. Yim, Y.J. Kim, J.H. Ko, Y.E. Cho, S.M. Kim, J.Y. Kim, S. Lee, and J.H. Park. The putative tumor suppressor gene GLTSCR2 induces PTEN-modulated cell death. *Cell Death Differ*, 14(11):1872–1879, Nov 2007.

217. J.C. Yoon, A.J. Ling, M. Isik, D.Y. Lee, M.J. Steinbaugh, L.M. Sack, A.N. Boduch, T.K. Blackwell, D.A. Sinclair, and S.J. Elledge. GLTSCR2/PICT1 links mitochondrial stress and Myc signaling. *Proc Natl Acad Sci U S A*, 111(10):3781–3786, Mar 2014.

218. T.Y. Yoon, X. Lu, J. Diao, S.M. Lee, T. Ha, and Y.K. Shin. Complexin and Ca2+ stimulate SNARE-mediated membrane fusion. *Nat Struct Mol Biol*, 15(7):707–713, Jul 2008.

219. H. Yu, R.F. Pretot, T.R. Burglin, and P.W. Sternberg. Distinct roles of transcription factors EGL-46 and DAF-19 in specifying the functionality of a polycystin-expressing sensory neuron necessary for *C. elegans* male vulva location behavior. *Development*, 130(21):5217–5227, Nov 2003.

220. E. Zacharioudaki, S.S. Magadi, and C. Delidakis. bHLH-O proteins are crucial for Drosophila neuroblast self-renewal and mediate Notch-induced overproliferation. *Development*, 139(7):1258–1269, Apr 2012.

221. C. Zhang, Y.S. Kho, Z. Wang, Y.T. Chiang, G.K. Ng, P.C. Shaw, Y. Wang, and R.Z. Qi. Transmembrane and coiled-coil domain family 1 is a novel protein of the endoplasmic reticulum. *PLoS One*, 9(1):e85206, 2014.

222. P. Zhang, X. Ma, E. Song, W. Chen, H. Pang, D. Ni, Y. Gao, Y. Fan, Q. Ding, Y. Zhang, and X. Zhang. Tubulin cofactor a functions as a novel positive regulator of ccRCC progression, invasion and metastasis. *Int J Cancer*, 133(12):2801–2811, Dec 2013.

223. X. Zhang, C.C. Milton, C.L. Poon, W. Hong, and K.F. Harvey. Wbp2 cooperates with Yorkie to drive tissue growth downstream of the Salvador-Warts-Hippo pathway. *Cell Death Differ*, 18(8):1346–1355, Aug 2011.
